# Digital Technologies for Health Promotion and Disease Prevention in Older People: Scoping Review

**DOI:** 10.2196/43542

**Published:** 2023-03-23

**Authors:** Karina Karolina De Santis, Lea Mergenthal, Lara Christianson, Annalena Busskamp, Claudia Vonstein, Hajo Zeeb

**Affiliations:** 1 Department of Prevention and Evaluation Leibniz Institute for Prevention Research and Epidemiology Bremen Germany; 2 Leibniz-Science Campus Digital Public Health Bremen Bremen Germany; 3 Department of Administration Leibniz Institute for Prevention Research and Epidemiology Bremen Germany; 4 Department Q6- Adults, Seniors, Women’s and Men’s Health, Health Equity Federal Centre for Health Education Cologne Germany; 5 Faculty 11 Human and Health Sciences University of Bremen Bremen Germany

**Keywords:** digital technology, health technology, digital public health, health promotion, disease prevention, healthy aging, elderly population, older adult, older population, scoping review

## Abstract

**Background:**

Digital technologies have the potential to contribute to health promotion and disease prevention in the aging world.

**Objective:**

This study aims to identify digital technologies for health promotion and disease prevention that could be used independently by older people in nonclinical settings using a scoping review.

**Methods:**

Through database (MEDLINE, PsycINFO, CINAHL, and SCOPUS; to March 3, 2022) and manual searches (to June 14, 2022), 90 primary studies and 8 systematic reviews were included in this scoping review. The eligibility was based on the PCC (Population, Concept, and Context) criteria: (1) people aged 50 years or older (population), (2) any digital (health) technology (eg, smartphone apps, websites, virtual reality; concept), and (3) health promotion and disease prevention in daily life in nonclinical and noninstitutional settings (context). Data items included study characteristics, PCC criteria, opportunities versus challenges, and evidence gaps. Data were synthesized using descriptive statistics or narratively described by identifying common themes.

**Results:**

The studies were published in 2005-2022 and originated predominantly from North America and Europe. Most primary studies were nonrandomized, reported quantitative data, and investigated effectiveness or feasibility (eg, acceptance or usability) of digital technologies in older people. The participants were aged 50 years to 99 years, predominantly female, affluent (ie, with high income, education, and digital competence), and intended to use or used digital technologies for a median of 3 months independently at home or in community settings. The digital technologies included mobile or nonmobile technologies or virtual reality. The studies used “modern devices” (eg, smartphones, wearables, or gaming consoles) or modern and “older devices” (eg, computers or mobile phones). The users interacted with digital technologies via websites, emails, text messages, apps, or virtual reality. Health targets of digital technologies were mobility, mental health, nutrition, or cognition. The opportunities versus challenges of digital technologies were (1) potential health benefits versus unclear or no benefits for some outcomes, (2) monitoring of health versus ethical issues with data collection and management, (3) implications for functioning in daily life (ie, potential to prolong independent living) versus unclear application for clinical management or care, (4) tailoring of technical properties and content toward older users versus general use, (5) importance of human support for feasibility versus other factors required to improve feasibility, (6) reduction of social isolation versus access to digital technologies, and (7) improvement in digital competence versus digital divide.

**Conclusions:**

Various digital technologies were independently used by people aged 50 years or older for health promotion and disease prevention. Future studies should focus on (1) more diverse populations of older people, (2) new digital technologies, (3) other (clinical and care) settings, and (4) outcome evaluation to identify factors that could enhance any health benefits of digital technologies.

**International Registered Report Identifier (IRRID):**

RR2-10.2196/37729

## Introduction

Digital technologies have the potential to contribute to health promotion and disease prevention [1]. However, the majority of commercially available digital platforms focus on management of existing diseases rather than on health promotion and disease prevention [2]. Furthermore, predominantly younger and more affluent people (ie, those with more education, higher income, and higher digital competence) tend to use and possibly benefit from digital technologies that support healthy behavior [3].

Older people could especially benefit from interventions addressing health promotion and disease prevention (for review, see [4]). Although most interventions targeting this population are analog (ie, nondigital) [5], digital technologies to support specific health outcomes, such as physical activity in people older than 50 years have already been identified in other reviews (eg, [6,7]). One advantage of interventions supported by digital technologies is the potential for using such technologies independently at home. However, declining motor and cognitive functioning could contribute to various barriers associated with the use of digital technologies by older people [8]. Furthermore, the trust in digital competence of older people is low in that older people are less likely than younger people to receive access to any digital health services by their health care providers [9]. This is despite older people reporting that they are willing to engage with new technologies [9] and maintain such engagement for longer than the general population [10].

**Figure 1:**
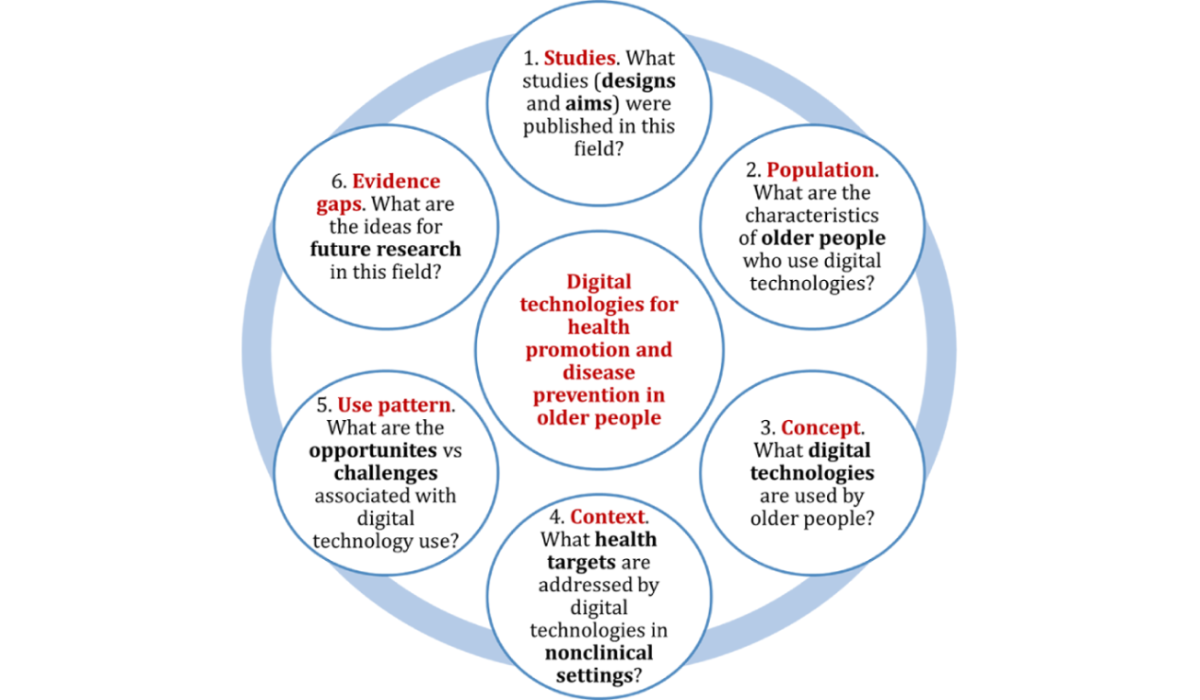
Objectives of this scoping review.

This study aimed to identify digital technologies for health promotion and disease prevention that could be used independently by older people in nonclinical settings using a scoping review. A scoping review methodology was used due to the broad scope of the population (older people), the concept (any digital technologies), and the context (health promotion and disease prevention in nonclinical settings). This scoping review was guided by the Arksey and O’Malley [11] framework for scoping studies. The objectives of this scoping review were to describe (1) studies published in this field so far (designs and aims), (2) characteristics of older people who independently use digital technologies, (3) digital technology (ie, types, devices, and use purpose), (4) health targets (eg, mobility), (5) digital technology use pattern (eg, setting, use duration, adherence, and opportunities and challenges associated with use), and (6) evidence gaps in this field (Figure 1).

## Methods

### Study Design

This study used a scoping review design and adhered to the PRISMA-ScR (Preferred Reporting Items for Systematic Reviews and Meta-Analyses Extension for Scoping Reviews) checklist [12] (Table S1 in Multimedia Appendix 1).

### Protocol and Registration

A protocol for this scoping review was prospectively registered [13] and published [4]. There were no changes between the published protocol [4] and this scoping review.

### Eligibility Criteria

The eligibility for our scoping review was based on the PCC (Population, Concept, and Context) criteria (Textbox 1; Textbox S1 in Multimedia Appendix 1).

**Inclusion criteria for this scoping review.**

**Population**

- Older people (all participants aged 50 years or above)
- Any health status (healthy, at risk for any disease, or with any disease)

**Concept**

- Digital (health) technologies: (1) eHealth (information and communication technology to support health) and (2) mobile health (mHealth; digital devices with mobile and wireless technologies to support health objectives) [14]
- Digital devices: any “older technologies” (eg, computers or mobile phones) or “modern technologies” (eg, smartphones, wearables, or gaming consoles)

**Context**

- Health promotion and disease prevention defined as any measures used to maintain or improve the existing health status and prevent the onset of new diseases
- Any health target in the context of healthy aging (eg, mobility, nutrition, cognition, or mental health)
- Any nonclinical and noninstitutionalized setting (eg, recruited from a community or living independently at home)

**Study type**

- Primary studies with any designs (randomized or nonrandomized) or data type (quantitative, qualitative, or mixed)
- Reviews with systematic methodology (eg, systematic reviews)
- Studies published as papers in peer-reviewed journals in English, German, or French and available as full text (other languages may be included if assistance from native speakers at our institutions was available)

The inclusion of other reviews in this scoping review was based on 2 reasons. First, we aimed to identify the relevant literature on the 3 broad topics (digital technologies, health promotion and disease prevention, and older people) either in our literature search or in other reviews. Second, we aimed to provide an overview of existing reviews to potentially reduce research waste that occurs when new reviews are produced despite the existence of other reviews on similar topics [15,16].

According to our protocol [4], we planned to include studies with older people using any age range as defined by study authors. However, due to substantial heterogeneity in terminology used to define older people, the studies selected for this scoping review included people aged 50 years or above because such an age is considered as the onset of older age [17].

### Information Sources

The information sources for this scoping review were bibliographic databases, bibliographies of any included systematic reviews, Google Scholar, and most relevant journals in the field of digital health [18]. The databases were chosen based on our institutional access and because they identified relevant literature in our other searches for digital health technologies. Due to potential financial interests in the field of digital health technologies, only peer-reviewed literature was included, and conflicts of interest statements were assessed per article. We assumed that such peer-reviewed literature may critically and objectively evaluate the health applications of digital technologies in older people.

### Search Strategy

The search strategy was developed and calibrated by the team. The electronic search was performed by a librarian on our team (LC) in MEDLINE, PsycINFO, CINAHL, and SCOPUS from inception through to March 3, 2022. The search syntax included the terms “older adults” AND “digital technologies” AND (“health promotion” OR “disease prevention”) in titles, abstracts, or subject terms (Multimedia Appendix 2). Manual searches of bibliographies, Google Scholar, and other relevant journals were performed by 3 authors (KKDS, LC, LM) up to June 14, 2022 (Table S2 in Multimedia Appendix 1). All search results were exported and managed in EndNote X9 (Clarivate).

### Study Selection

Screening based on title, abstract, and full text was performed in EndNote independently by any 2 authors, and consensus was reached by discussion (Figure S1 and Table S3 in Multimedia Appendix 1).

### Data Charting

A data charting form was developed in Excel (version 10; Microsoft Corp; Multimedia Appendix 3) and calibrated within the team. Data charting was performed independently by any 2 researchers, and consensus was reached by discussion.

We performed data charting by extracting quantitative data and qualitative author statements from studies. The quantitative data were directly coded into predefined categories (eg, participant characteristics). Charting of qualitative information in scoping reviews involves sorting such data into meaningful categories or themes [11]. According to recommendations for scoping reviews [19], we first coded the relevant statements from studies (eg, noted the study conclusion according to authors). We then classified such statements into themes based on semantic analysis (eg, we detected opportunities of digital technologies in author statements) or latent analysis (eg, we detected opportunities of digital technologies that were not explicitly stated by study authors but inductively emerged from the study conclusion).

### Data Items

A list of data items (Textbox 2) was developed based on the objectives (Figure 1) for this scoping review, and data coding instructions were summarized in a coding manual (Table S4 in Multimedia Appendix 1). Since digital technologies are typically described using heterogeneous terminology [15], we used 3 items to capture the different aspects of such technologies (technology type, device type, and interaction between the user and technology).

**Data items in this scoping review.**

1. Bibliographic information: publication year, author region, funding sources
2. Study designs and aims: randomized or nonrandomized, data type (eg, quantitative), overlap in primary studies in reviews, aims (eg, effectiveness or feasibility: ie, acceptance, usability, engagement, satisfaction, or adherence)
3. Population (older people): sample size, data collection region, age, gender, health, employment status, socioeconomic status (based on income and education), digital competence
4. Concept (digital technology): type (eg, mobile technology with internet access), device (eg, computer), interaction with digital technology (eg, via website)
5. Context (health promotion and disease prevention): health target (eg, mobility), health purpose (eg, monitoring)
6. Use pattern of digital technologies: setting (eg, community), use duration, adherence, opportunities, challenges
7. Evidence gaps: ideas for future research

### Critical Appraisal

A critical appraisal of individual primary studies is typically not performed in scoping reviews. The quality of existing evidence was indirectly assessed based on study designs included in this scoping review. The critical appraisal of systematic reviews was performed with AMSTAR 2 (A Measurement Tool to Assess Systematic Reviews, version 2) [20]. AMSTAR 2 generates the overall confidence rating in the results of a systematic review (critically low, low, moderate, or high) that indicates if systematic reviews have any weaknesses in their methodology and interpretation of results. The appraisals with AMSTAR 2 were performed independently by 2 authors, and consensus was reached by discussion. The number of critical weaknesses and the overall confidence rating for each systematic review were coded into a spreadsheet (Excel, version 10; Multimedia Appendix 4).

### Synthesis of Results

The quantitative data items and AMSTAR 2 appraisal outcomes were synthesized using descriptive statistics (frequencies or means, SDs, or ranges) in SPSS 24 (IBM Corp). The qualitative data items were synthesized narratively into themes. Each theme was mentioned in at least one study. Since we aimed to scope the field, we did not weigh the importance of the themes (eg, by counting the number of times each theme was mentioned in all studies).

## Results

### Study Selection

Based on our electronic and manual searches, 2188 sources were screened, and study selection was summarized in a flowchart (Figure S1 in Multimedia Appendix 1). A total of 90 primary studies (reported in 105 publications) [9,10,21-123] and 8 systematic reviews [124-131] were included in this scoping review (for the list of excluded studies, see Table S3 in Multimedia Appendix 1). All results are reported in Textbox S2 in Multimedia Appendix 1.

### Bibliographic Characteristics of Included Studies

The primary studies were published from 2005 to 2022 (Table 1). There was an exponential increase in the number of published studies indicating a growing interest in this area of research over time (Table 1). Most studies originated from North America and Europe (74/90), followed by Asia and Australia (16/90). Conflict of interest due to funding was absent (76/90) or unclear (4/90), or funding was not reported (10/90).

**Table 1:** Publication years of 105 publications reporting the results of 90 primary studies included in this scoping review.

Publication year	Number of publications, n
2005	2
2006	0
2007	1
2008	2
2009	2
2010	1
2011	1
2012	2
2013	9
2014	7
2015	10
2016	12
2017	4
2018	10
2019	12
2020	12
2021	16
2022	2

### Objective 1 (Studies): Study Designs and Aims

**Figure 2:**
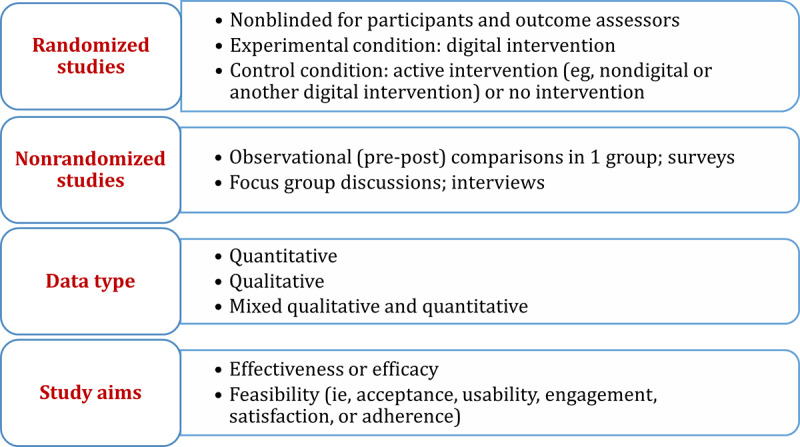
Study designs and aims.

Most primary studies were nonrandomized (50/90), reported quantitative data (75/90), and investigated effectiveness (61/90) or feasibility (53/90) of digital technologies in older people (Figure 2).

### Objective 2 (Population): Older People as Users of Digital Technologies

**Figure 3:**
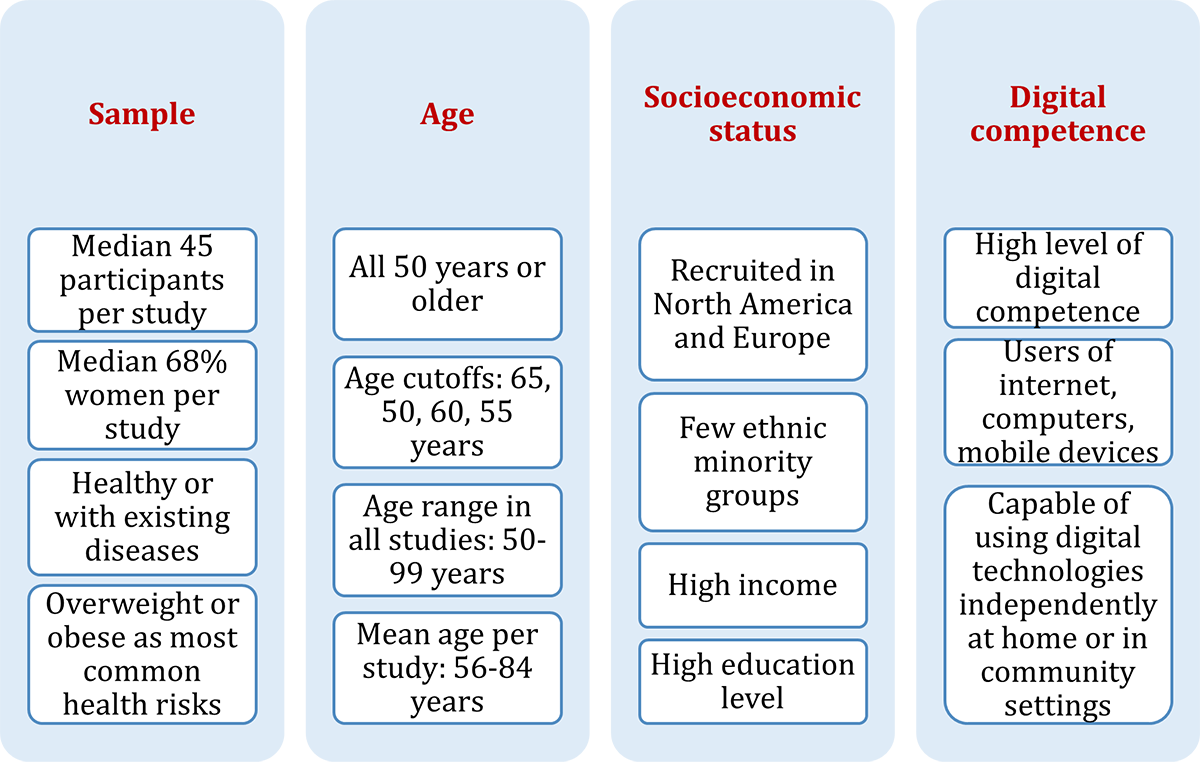
Older people as users of digital technologies.

The primary studies included a median of 45 participants who were predominantly female and either healthy or at risk for or with existing diseases (Figure 3). The age of older people was highly variable in all studies (from 50 years to 99 years). The most common age cutoffs were (1) 65 years (23/90), (2) 50 years (18/90), (3) 60 years (16/90), and (4) 55 years (15/90).

Most participants were recruited in North America and Europe (75/90). Among studies with information on the socioeconomic status, most studies reported high income (25/28) and high education level (55/65) for most study participants. Some participants were employed (7/90). Only 4 of 90 studies focused on ethnic minority groups.

If reported, digital competence was high in most studies (62/67) based on self-reported use of the internet and digital devices, such as computers or mobile phones, in daily life. All participants lived independently (ie, were not institutionalized at clinical or care institutions) and were capable of using the digital technologies at home (with or without human support provided by study staff).

### Objective 3 (Concept): Digital Technologies Used by Older People

**Figure 4:**
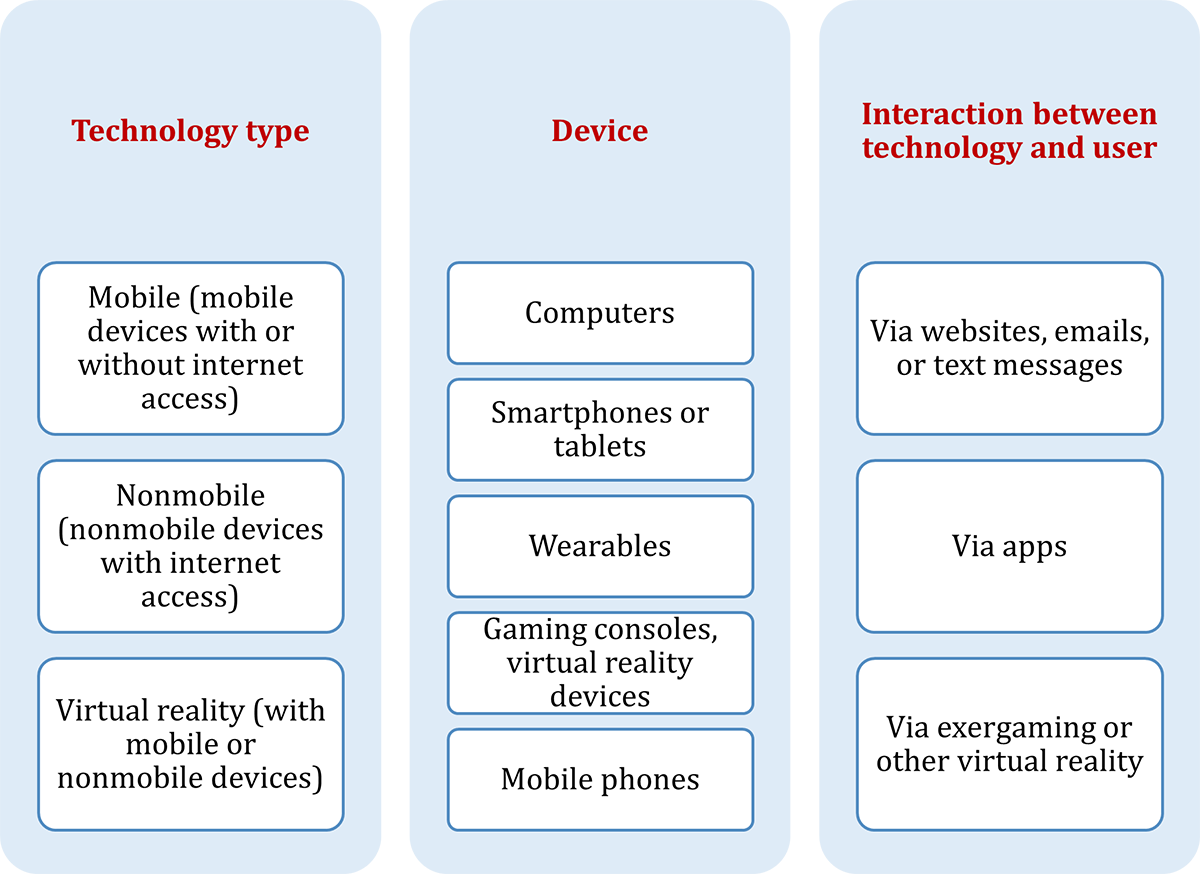
Digital technologies used by older people.

Digital technologies were described in all studies using heterogeneous terminology. We classified the digital technologies into (1) “mobile technologies” (with or without internet access; 39/90), (2) “nonmobile technologies” (with internet access; 33/90), and (3) “virtual reality” (17/90) or other unspecified technologies (2/90; Figure 4). We classified the devices used in studies as “modern devices” (eg, smartphones, tablets, wearables, or gaming consoles; 46/90) or a mix of “older devices” (eg, computers or mobile phones) and “modern devices” (42/90). The most commonly used devices were (1) computers (33/90), (2) smartphones or tablets (25/90), (3) wearables (21/90), (4) gaming consoles or other virtual reality devices (eg, camera, step pad, headset, or robot; 16/90), and (5) mobile phones or iPods (9/90). The interaction of older people with digital technologies occurred via (1) websites, emails, or text messages (38/90); (2) apps (37/90); or (3) exergaming or other virtual reality (18/90).

### Objective 4 (Context): Health Targets of Digital Technologies

**Figure 5:**
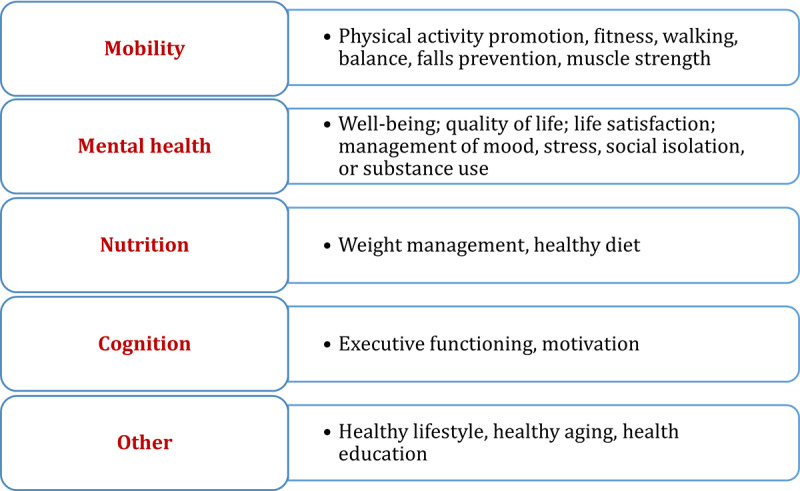
Health targets of digital technologies used by older people.

The most commonly reported health targets of digital technologies were (1) mobility (72/90), (2) mental health (17/90), (3) nutrition (15/90), (4) cognition (7/90), and (5) other unspecified measures to promote healthy aging (10/90; Figure 5). There were several health purposes of using digital technologies that were either reported in studies or inductively emerged based on study description. Digital technologies were used to provide feedback on performance; encourage or measure engagement (eg, based on the login data); monitor or track health; provide reminders; provide recommendations and educational information regarding healthy lifestyle; and encourage goal setting, motivation, and social networking or support.

### Objective 5 (Use Pattern): Opportunities and Challenges With Digital Technologies

**Figure 6:**
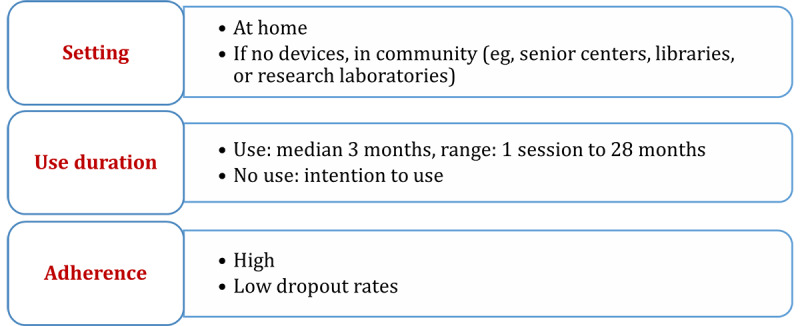
Use pattern of digital technologies.

Digital technologies were used independently at home or in community settings for a median of 3 months (82/90; Figure 6). Intention to use digital technologies was investigated in 8 of 90 studies. If reported, adherence to use of digital technologies was high (ie, dropout rates per study were less than 50% in 61 of 67 studies).

There were several opportunities and challenges associated with digital technology use that were either reported in studies or inductively emerged based on study description (Table 2). The opportunities versus challenges of digital technologies were (1) potential health benefits versus unclear or no benefits for some outcomes, (2) monitoring of health versus ethical issues with data collection and management, (3) implications for functioning in daily life (ie, potential to prolong independent living) versus unclear application for clinical management or care, (4) tailoring of technical properties and content toward older users versus general use, (5) importance of human support for feasibility versus other factors required to improve feasibility, (6) reduction of social isolation versus access to digital technologies, and (7) improvement in digital competence versus digital divide.

**Table 2:** Opportunities and challenges with digital technologies.

Topics	Opportunities	Challenges
Potential health benefits	Potential (small) health benefits exist for objectively measured outcomes (eg, physical activity), relative to baseline or to no intervention and in the short term (ie, pre versus post-digital technology use); digital technologies can be included in complex interventions (eg, audiobooks to promote walking, apps with local walking trails or healthy food outlets).	Unclear or no benefits for outcomes that are difficult to measure objectively (eg, well-being), relative to active interventions (eg, group exercise) and in the long term; complex interventions can be too time-consuming.
Monitoring of health	Monitoring and tracking of own health useful	Ethical issues in detecting own decline in functioning and in data management (eg, who has access to and how data will be used)
Implications for functioning in daily life	Even small benefits could improve functioning in daily life (eg, walking), prolong independent living, and provide access to previously enjoyed activities (eg, bowling via exergaming).	Unclear application for clinical management or care
Tailoring toward older users	Devices and their content should be developed with and for older people. Relevance to daily life and perception of health benefits need to be tailored toward the users.	User experience depends on technical properties (eg, button size or interface complexity) and is reduced by inappropriate content (eg, too high goal settings for physical activity).
Understanding that feasibility depends on human support	Feasibility (ie, acceptance, usability, engagement, satisfaction, or adherence) can be improved by human support from study staff (eg, technical support, reminders, and assistance with the health content) or other study participants (eg, via social networks, such as walking groups or online discussion boards).	Feasibility under real-life conditions (ie, alone without any human support) decreases over time; potentially high costs of human support; feasibility depends on multiple factors (eg, age, gender, digital competence and interest, health status and competence, education, duration and frequency of use, and reminders).
Reduction of social isolation	Reduced social isolation by improved assess for geographically or functionally isolated people (eg, people with low mobility)	Devices may not be available for independent use at home (eg, gaming consoles or stable internet access).
Improvement in digital competence	Improvement in digital competence in daily life (eg, computer operating skills, using videocalls)	Digital divide: higher use and potential benefits for more affluent, educated, and digitally competent people

### Objective 6 (Evidence Gaps): Ideas for Future Research

There were several evidence gaps that were either reported in studies or inductively emerged based on study description (Textbox 3). Future studies should focus on (1) more diverse populations of older people, (2) new digital technologies, (3) other (clinical and care) settings, and (4) outcome evaluation to identify factors that could enhance any health benefits of digital technologies.

**Evidence gaps.**

**Population**

- Investigating other (more diverse) populations based on (1) socioeconomic status (less educated, less affluent), (2) health status (less healthy, more sedentary, with worse cognitive and mental functioning), (3) psychosocial status (less motivated, more socially isolated), (4) culture (ethnicity, cultural background, language), (5) digital competence (less competent)

**Concept**

- Development of new digital health technologies and investigating their mechanisms of action (ie, how they work)
- Tailoring the digital technologies toward individual needs of participants
- Assessing the cost-effectiveness of digital technologies

**Context**

- Investigating other contexts and settings (eg, application of data for clinical management and in care settings)

**Outcomes**

- Evaluation of effectiveness (health benefits in the short versus long term or among different digital technologies)
- Identifying factors that improve the effectiveness and feasibility of digital technologies (eg, factors that positively influence user experience)

### Overview of 8 Systematic Reviews

In addition to the primary studies, we identified 8 relevant systematic reviews from electronic and manual searches (Table S3 in Multimedia Appendix 1). Despite a common focus on physical activity promotion with digital technologies in older people, the reviews included different primary studies (Textbox S3 in Multimedia Appendix 1). Among 61 primary studies included in all 8 systematic reviews, most primary studies (38/61) were included in only 1 systematic review, and only 17 of 61 primary studies were also included in this scoping review (either from electronic or manual searches). Further inspection revealed that some of the 61 primary studies did not focus on digital technologies or focused on other contexts or settings (eg, clinical patient management or participant recruitment from care facilities).

The reviews reported that digital technologies tended to improve physical activity outcomes relative to baseline or to no intervention in older people. No changes or worsening in physical activity were reported relative to other active interventions (digital or nondigital).

According to AMSTAR 2, the confidence in the results of the systematic reviews was either low (3/8) or critically low (5/8; Multimedia Appendix 4). There were 1 to 4 critical weaknesses in each systematic review. The most common weaknesses were (1) no review protocol, (2) no list of excluded studies, and (3) no report of sources of funding for primary studies included in reviews.

## Discussion

### Principal Findings

This scoping review, based on data from 90 primary studies and 8 systematic reviews, showed that people aged 50 years or older can independently use various digital technologies designed to promote healthy behavior. The population of older users of digital technologies was highly heterogeneous in terms of age (ie, ranging from younger, regular users to older, first-time users of digital technologies) and included predominantly female and affluent people (ie, more educated and wealthier). Device types used in the studies reflect the enormous technological progress of the last 20 years. About one-half of all studies relied on older technologies, such as websites accessed via computers or text messages sent to mobile phones, while another one-half included modern mobile devices (eg, smartphones, tablets, or wearables) or gaming consoles. Human support was important for feasibility (ie, acceptance, usability, engagement, adherence, or satisfaction). Digital technology use declined under real-life conditions (ie, when used alone without human support), if the health relevance of digital technologies was not explicitly evident, and if devices or their content were not tailored toward the needs of older people. Most studies investigated mobility, while other health targets, such as mental health, nutrition, cognition, and a general healthy lifestyle, were less commonly investigated, possibly due to difficulties in objective measurement of such outcomes.

### Comparison With Prior Work

The studies included in this scoping review show the remarkably fast technological advancement from 2005 until 2022. Some barriers related to digital technology use mentioned in the earlier studies are no longer relevant. For example, access to personal computers at home was low, especially in less affluent populations in the older studies. Meanwhile access to personal computers at home might be *once again* low due to the availability of mobile technologies, including smartphones and tablets that tended to be included among devices for accessing the internet in the newer studies. The pattern of internet use has also rapidly changed over this short period of time, from occasional use per week in the older studies to continuous use throughout the day by 2022. Furthermore, the need to manually enter data can be avoided because modern technologies, such as smartphones or smartwatches, can automatically detect and measure some functions, such as physical activity or cardiovascular fitness.

Although older people are willing to use digital technologies for health promotion, various facilitators are required to further encourage digital technology use [8,132,133]. One important facilitator appears to be human support. According to our scoping review, such support includes (1) continuous technical support on the phone or onsite (ie, a visit at home), (2) human coaching (eg, reminder calls, text messages, or emails from study staff to motivate the users), and (3) social networks established for older people either in real life or virtually (eg, support groups via online discussion boards). Furthermore, digital technologies need to be designed for and tailored toward the needs of older people, as already suggested a decade ago [124]. For example, a user-centered participatory approach should be used to design, develop, and evaluate digital technologies for older people [21,22], because effectiveness of any health program depends on positive user experience [125]. Such a positive user experience could be enhanced via (1) manageable complexity and costs of digital devices [126], (2) improved motivation and high enjoyment among participants [127], (3) consideration of the age-related skills to use new digital technologies [126], and the general experience with digital technologies [128,129] that may require human support [130]. Digital technologies for older people should be developed around the goal setting theory to provide explicit information on potential health benefits by including educational content, reminders, and feedback [126].

Studies included in this scoping review show that ownership of digital devices or intentions to use them do not guarantee their actual use in the health context. In general, health benefits of digital technologies are unclear based on small differences in outcomes before versus after digital technology use and heterogenous outcomes assessed in studies. The most common health target of digital technologies for promotion of healthy behavior in any age group is physical activity [134]. This health target was also the most common in studies included in this scoping review. This is not surprising, since older people identify physical activity as the main domain of health promotion [23]. Physical activity can be objectively measured and monitored using modern mobile devices (eg, smartphones with GPS technology) that can be easily carried around without the need to attach them, like wearables [130]. Various digital technologies can contribute to promotion of physical activity, especially in the short term and relative to no intervention groups [128]. Although possibly not clinically meaningful, small changes in healthy behavior can positively affect daily functioning and overall well-being in terms of the improved ability to perform daily tasks. Such improvements could empower older people by prolonging independent living and promoting freedom [24], but this topic requires further research.

### Evidence Gaps and Ideas for Future Research

Future research is needed to determine the generalizability of the results in this scoping review. Future studies should focus on (1) more diverse populations of older people, (2) new digital technologies, (3) other (clinical and care) settings, and (4) outcome evaluation to identify factors that could enhance any health benefits of digital technologies. In general, evaluation of effectiveness of digital technologies is difficult due to the lack of standardized terminology, heterogeneous descriptions of devices and procedures involved in the implementation of digital technologies, and the need for new study designs that could be used in this rapidly evolving field [15]. These reasons and inadequate reporting contribute to a generally low confidence in the results of systematic reviews in the field of digital health [15]. The 90 primary studies in this scoping review also used heterogeneous designs and implementation strategies. Most studies were nonrandomized, while some did not include control groups or relied on self-reported data. Thus, future research should focus on the identifying factors that could enhance the effectiveness of digital technologies and promote their independent use in the longer term (ie, beyond the study duration). Such factors include the digital technology types (eg, those tailored toward older people that are easy to use, automatically collect data, and encourage use via feedback and reminders) and the nondigital elements in digital health interventions (eg, social networks among study participants that improve the motivation to continue using digital technologies in the longer term). Furthermore, future systematic reviews are needed to evaluate individual digital technologies or to compare the effects of different digital technologies on health outcomes in older people. Such systematic evaluation is needed for any stakeholder to provide advice and guidance or develop new technologies addressing health promotion and disease prevention that target the needs of older people.

There is yet much to learn about the use of digital technologies in the field of public health and focusing on older adults. Digital technologies can positively enable and transform the way interventions targeting health promotion and disease prevention are designed and implemented [135]. Digital public health interventions for older people should address essential public health functions relevant for this population through digital means and include members of the target population in the development process to improve social acceptance and achieve health benefits [136]. Future research on digital technologies addressing public health functions for older people could focus on various aspects of the 10 e’s framework of eHealth [137]. The framework was developed to define eHealth in the context of health care and the 10 e’s address various aspects of “e” in the term “eHealth” beyond “electronic” health [137]. Adapting this framework to the field of public health would mean that digital technologies for older people need to (1) be efficient at reducing health care costs by promoting healthy behavior, (2) enhance health and prevent disease, (3) be evidence-based according to a rigorous scientific evaluation, (4) empower users by making health knowledge accessible, (5) encourage shared decision-making in the health context (eg, using one’s own data to support clinical decision-making), (6) educate the users, (7) enable data and information exchange, (8) extend the scope of health promotion beyond analog boundaries (eg, use the virtual environment to promote health), (9) be ethical in terms of data sharing and privacy, and (10) promote equity at improving access to health promotion measures for those at need (eg, less affluent, less healthy, or less digitally competent).

### Limitations

This scoping review had several limitations. First, locating the relevant studies was surprisingly difficult despite carefully designed search syntax. Consequently, 38 of the 98 included studies were located from manual searches, and there was a low overlap in primary studies either among the relevant 8 systematic reviews or among the reviews and our search. It is likely that highly heterogeneous terminology used in the field of digital technologies for health promotion and disease prevention [15,134,138] contributed to difficulties in locating the relevant literature. Furthermore, it cannot be ruled out that some located studies were incorrectly excluded based on limited information regarding the setting. In general, studies were excluded if digital technologies were not used independently (eg, used by caregivers of older people or if we could not determine if the use was independent) or used in clinical treatment (eg, as part of disease management). Thus, the 98 studies included in this scoping review can be considered a meaningful but far from complete sample of the literature in this field.

Second, due to the focus on independent use, we included studies with samples representing younger old age that often included more affluent people. Such samples are more likely to be digitized and own digital devices for personal use. People from older age groups may require assistance with daily living, and less affluent people may not use digital technologies in the health context. The focus on younger groups of older adults is important to prepare these groups for health challenges of old age by using digital technologies to promote physical activity, reduce loneliness, and keep mentally and cognitively fit [125]. Future research is needed to promote the use of digital health technologies into older age and for all groups along the sociodemographic spectrum. Especially important is also development of strategies necessary to recruit older people from lower socioeconomic and ethnically diverse backgrounds.

Third, this scoping review did not investigate the effectiveness of digital technologies. For example, it is unclear if modern technologies (eg, smartphones) are better than older technologies (eg, computers) at promoting healthy behavior among older people. There are advantages of both technology types. Some older people may be more familiar and more likely to own and use older technologies (eg, mobile phones without internet access). Modern technologies (eg, smartphones) are useful for automatic data collection, but their operation may be difficult due to small screen size or buttons, the need to operate a touch screen, or difficulties in attaching the device (eg, a smartwatch). Although computers were the most technologically advanced devices available for personal use in the older studies, the newer studies incorporated various devices in their digital interventions (eg, any device with internet access).

Finally, it was difficult to determine the commercial interests in the included studies. A common weakness of systematic reviews of digital technologies is that they do not report the sources of funding in primary studies [15]. Our scoping review shows that 14 of the 90 primary studies either failed to report funding or the reported conflict of interest due to funding was unclear. Any potential conflicts of interest arising from commercial interests in the field of digital health technologies need to be carefully reported in primary studies and assessed by review authors.

### Dissemination

The results of this study were presented in a conference poster (15th European Public Health Conference, November 2022, Berlin, Germany [139]), will be disseminated in English through this article, and will be shared in German through a project report. Furthermore, we summarized the main results for the nonscientific audience using plain language summaries in form of infographics in English and in German (Multimedia Appendix 5 and Multimedia Appendix 6, respectively).

### Conclusions

Various digital technologies were independently used by people aged 50 years or older for health promotion and disease prevention. The digital technologies were modern (eg, smartphones) or older (eg, computers), and the interaction with such technologies occurred via different methods (eg, websites, emails, text messages, apps, or virtual reality). Different health targets were addressed (ie, mobility, mental health, nutrition, and cognition). Although digital technologies could contribute to health benefits, the challenges associated with their use need to be considered. Future studies should focus on (1) more diverse populations of older people, (2) new digital technologies, (3) other (clinical and care) settings, and (4) outcome evaluations to identify factors that could enhance any health benefits of digital technologies.

## Appendix

Multimedia Appendix 1 Supplementary information.

Multimedia Appendix 2 Search strategy and results.

Multimedia Appendix 3 Data file.

Multimedia Appendix 4 A Measurement Tool to Assess Systematic Reviews, version 2 (AMSTAR 2) ratings.

Multimedia Appendix 5 Plain language summary (English).

Multimedia Appendix 6 Plain language summary (German).

## Abbreviations

AMSTAR 2: A Measurement Tool to Assess Systematic Reviews, version 2
PCC: Population, Concept, and Context
PRISMA-ScR: Preferred Reporting Items for Systematic Reviews and Meta-Analyses Extension for Scoping Reviews
