# Supplementary material for: Digital Technologies for Health Promotion and Disease Prevention in Older People: Scoping Review
Source: J Med Internet Res. 2023 Mar 23;25:e43542. doi: 10.2196/43542 (PMC10131689; doi:10.2196/43542)
Supplement: Multimedia Appendix 1 [file jmir_v25i1e43542_app1.docx]

Multimedia Appendix 1

**Digital Technologies for Health Promotion and Disease Prevention in Older People: Scoping Review**

Karina Karolina De Santis (1,2), PhD; Lea Mergenthal (1), BA; Lara Christianson (3), MLS; Annalena Busskamp (4); MSc, Claudia Vonstein (4), MA; Hajo Zeeb (1,2,5), MD, PhD, Prof. Dr.

Content

[Table S1. PRISMA-ScR Checklist. 2](#_Toc120288102)

[Textbox S1. Inclusion and exclusion criteria. 5](#_Toc120288103)

[Table S2. Summary of the search strategy. 6](#_Toc120288104)

[Figure S1. Study selection (PRISMA [Preferred Reporting Items for Systematic Reviews and Meta-Analyses] flowchart). 7](#_Toc120288105)

[Table S3. List of included and excluded studies. 8](#_Toc120288106)

[Table S4. Data coding manual. 9](#_Toc120288107)

[Textbox S2. Results in 90 primary studies. 12](#_Toc120288108)

[Bibliographic Characteristics of Included Studies 12](#_Toc120288109)

[Objective 1 (Studies): Study Designs and Aims 14](#_Toc120288110)

[Objective 2 (Population): Older People as Users of Digital Technologies 14](#_Toc120288111)

[Objective 3 (Concept): Digital Technologies Used by Older People 19](#_Toc120288112)

[Objective 4 (Context): Health Targets of Digital Technologies 20](#_Toc120288113)

[Objective 5 (Use Pattern): Opportunities and Challenges with Digital Technologies 23](#_Toc120288114)

[Objective 6 (Evidence Gaps): Ideas for Future Research 38](#_Toc120288115)

[Textbox S3. Overlap among primary studies. 43](#_Toc120288116)

[References 44](#_Toc120288117)

## Table S1. PRISMA-ScR Checklist.

| **Section** | **Item** | **PRISMA-ScR Checklist Item** | **Fulfilled / Location** |
| --- | --- | --- | --- |
| Title | 1 | Identify the report as a scoping review. | yes |
| **Abstract** |  |  |  |
| Structured summary | 2 | Provide a structured summary that includes (as applicable): background, objectives, eligibility criteria, sources of evidence, charting methods, results, and conclusions that relate to the review questions and objectives. | yes |
| **Introduction** |  |  |  |
| Rationale | 3 | Describe the rationale for the review in the context of what is already known. Explain why the review questions/objectives lend themselves to a scoping review approach. | yes |
| Objectives | 4 | Provide an explicit statement of the questions and objectives being addressed with reference to their key elements (e.g., population or participants, concepts, and context) or other relevant key elements used to conceptualize the review questions and/or objectives. | Figure 1 |
| **Methods** |  |  |  |
| Protocol and registration | 5 | Indicate whether a review protocol exists; state if and where it can be accessed (e.g., a Web address); and if available, provide registration information, including the registration number. | yes |
| Eligibility criteria | 6 | Specify characteristics of the sources of evidence used as eligibility criteria (e.g., years considered, language, and publication status), and provide a rationale. | Textbox 1  Textbox S1 |
| Information sources | 7 | Describe all information sources in the search (e.g., databases with dates of coverage and contact with authors to identify additional sources), as well as the date the most recent search was executed. | Table S2 |
| Search | 8 | Present the full electronic search strategy for at least 1 database, including any limits used, such that it could be repeated. | Multimedia Appendix 2 |
| Selection of sources of evidence | 9 | State the process for selecting sources of evidence (i.e., screening and eligibility) included in the scoping review. | Figure S1, Table S3 |
| Data charting process | 10 | Describe the methods of charting data from the included sources of evidence (e.g., calibrated forms or forms that have been tested by the team before their use, and whether data charting was done independently or in duplicate) and any processes for obtaining and confirming data from investigators. | yes |
| Data items | 11 | List and define all variables for which data were sought and any assumptions and simplifications made. | Textbox 2  Table S4 |
| Critical appraisal of individual sources of evidence | 12 | If done, provide a rationale for conducting a critical appraisal of included sources of evidence; describe the methods used and how this information was used in any data synthesis (if appropriate). | yes |
| Summary measures | 13 | *Not applicable for scoping reviews* | *-* |
| Synthesis of results | 14 | Describe the methods of handling and summarizing the data that were charted. | yes |
| Risk of bias across studies | 15 | *Not applicable for scoping reviews* | *-* |
| Additional analyses | 16 | *Not applicable for scoping reviews* | *-* |
| **Results** |  |  |  |
| Selection of sources of evidence | 17 | Give numbers of sources of evidence screened, assessed for eligibility, and included in the review, with reasons for exclusions at each stage, ideally using a flow diagram. | Figure S1 |
| Characteristics of sources of evidence | 18 | For each source of evidence, present characteristics for which data were charted and provide the citations. | Multimedia Appendix 3 |
| Critical appraisal within sources of evidence | 19 | If done, present data on critical appraisal of included sources of evidence (see item 12). | Multimedia Appendix 4 |
| Results of individual sources of evidence | 20 | For each included source of evidence, present the relevant data that were charted that relate to the review questions and objectives. | Textbox S2-S3 |
| Synthesis of results | 21 | Summarize and/or present the charting results as they relate to the review questions and objectives. | Figure 2-6  Textbox 3  Table 2  Textbox S2-S3 |
| Risk of bias across studies | 22 | *Not applicable for scoping reviews* | *-* |
| Additional analyses | 23 | *Not applicable for scoping reviews* | *-* |
| **Discussion** |  |  |  |
| Summary of evidence | 24 | Summarize the main results (including an overview of concepts, themes, and types of evidence available), link to the review questions and objectives, and consider the relevance to key groups. | yes |
| Limitations | 25 | Discuss the limitations of the scoping review process. | yes |
| Conclusions | 26 | Provide a general interpretation of the results with respect to the review questions and objectives, as well as potential implications and/or next steps. | yes |
| **Funding** | 27 | Describe sources of funding for the included sources of evidence, as well as sources of funding for the scoping review. Describe the role of the funders of the scoping review. | yes |

‘Yes’ means that the item was addressed in our scoping review under the same subheading as the item in the checklist.

Source: Tricco AC, Lillie E, Zarin W, O'Brien KK, Colquhoun H, Levac D, et al. Prisma Extension For Scoping Reviews (Prisma-Scr): Checklist and explanation. Ann Intern Med. 2018;169(7):467-73. doi: 10.7326/m18-0850.

## Textbox S1. Inclusion and exclusion criteria.

| **Inclusion criteria**   1. Population: older people 2. Concept: digital health technologies 3. Context: health promotion and disease prevention 4. Setting: nonclinical (eg, daily life, home, and community) 5. Study type: primary studies with any designs or data type (quantitative and qualitative) and reviews with systematic methodology 6. Publication status: published in peer-reviewed journal 7. Publication language: English, German, or French (other language may be included if assistance from native speakers at our institutions is available) 8. Full-text accessible   **Exclusion criteria**   1. Older people not included 2. Digital health technologies not included 3. Other context than health promotion and disease prevention 4. Clinical setting (eg, aged care and clinical facility) 5. Other study types: protocols or narrative reviews 6. Other publication status: published without peer-review, dissertations, books, conference papers, comments, corrections, letters, and editorials 7. Publication language other than English, German, or French 8. Full-text not accessible |
| --- |

## Table S2. Summary of the search strategy.

| **Sources (time frame)** | **Simplified search syntax (Title OR Abstract OR Subject terms)** | **Studies n** |
| --- | --- | --- |
| **Databases** |  |  |
| MEDLINE through Ovid (from 1946 through to March 3, 2022) | “older adults” AND “digital technologies” AND (“health promotion” OR “disease prevention”) | 1165 |
| PsycINFO through Ovid (from 1806 through to March 3, 2022) | “older adults” AND “digital technologies” AND (“health promotion” OR “disease prevention”) | 243 |
| CINAHL through EBSCO (from 1981 through to March 3, 2022) | “older adults” AND “digital technologies” AND (“health promotion” OR “disease prevention”) | 1049 |
| SCOPUS (from 1981 through to March 3, 2022) | “older adults” AND “digital technologies” AND (“health promotion” OR “disease prevention”) | 501 |
| **Other** |  |  |
| Citation searches, Google Scholar, other relevant journal websites: Journal of Medical Internet Research (all journals), BMC Public Health, The Lancet Digital Health, PLOS Digital Health, Frontiers in Digital Health (from March 3 to June 14, 2022) | “older adults” AND “digital technologies” AND (“health promotion” OR “disease prevention”) | **38** |
| **Total (with duplicates)** |  | **2996** |
| **Total (without duplicates)** |  | **2188** |

## Figure S1. Study selection (PRISMA [Preferred Reporting Items for Systematic Reviews and Meta-Analyses] flowchart).

Records identified from citation searches, Google scholar, journal websites (n=38)

Records identified from databases (n=2958)

**Identification**

Duplicate records removed (n=808)

Records excluded (n=2004)

Exclusion 1: no older people (n=1439)

Exclusion 2: no digital technologies (n=301)

Exclusion 3: no promotion or prevention (n=167)

Exclusion 4: other context: clinical or care (n=24)

Exclusion 5: protocols, narrative reviews (n=41)

Exclusion 6: conference papers, books, dissertations, comments, corrections (n=32)

Records (titles and abstracts) screened by 2 authors (n=2188)

**Screening**

Records excluded (n=71)

Exclusion 1: no older people (n=16)

Exclusion 2: no digital technologies (n=8)

Exclusion 3: no promotion or prevention (n=17)

Exclusion 4: other context: clinical or care (n=27)

Exclusion 5: narrative review (n=1)

Exclusion 6: conference paper (n=2)

Records (full-text) screened by 2 authors (n=184)

**Eligibility**

90 primary studies (reported in 105 papers) and 8 systematic reviews included in this scoping review

**Included**

## Table S3. List of included and excluded studies.

| **Study status** | **Studies, n** | **Inclusion or exclusion** |
| --- | --- | --- |
| Total studies screened | 2188 |  |
| Include primary studies | 90 reported in 105 papers | [1-105] |
| Include reviews | 8 systematic reviews | [106-113] |
| Include from electronic search | 75 papers | [1, 6, 8-11, 13-15, 18, 20, 22-24, 26-28, 30-33, 35-38, 40-48, 53, 54, 56, 58-60, 62, 63, 65, 67-69, 71-75, 77, 78, 80, 81, 84-88, 90, 93, 94, 98, 101-105, 108-113] |
| Include from manual search | 38 papers | [2-5, 7, 12, 16, 17, 19, 21, 25, 29, 34, 39, 49-52, 55, 57, 61, 64, 66, 70, 76, 79, 82, 83, 89, 91, 92, 95-97, 99, 100, 106, 107] |
| Exclude studies full-text | 16 | E1: [114-129] |
|  | 8 | E2: [130-137] |
|  | 17 | E3: [138-154] |
|  | 27 | E4: [155-181] |
|  | 1 | E5: [182] |
|  | 2 | E6: [183, 184] |

**^b^**Exclusion criteria are shown in Textbox S1.

## Table S4. Data coding manual.

Checklist BEFORE coding the data:

- Inclusion 1: study in older adults (ALL 50 years or older)
- Inclusion 2: digital technology used as main device or mode of intervention delivery (exclude if landline telephones, pedometers or accelerometers used)
- Inclusion 3: health promotion and disease prevention (any focus)
- Inclusion 4: setting: daily life (at home), community (exclude if interventions performed in aged care or clinical facilities)

| **Bibliographic information** | |
| --- | --- |
|  | 1. First author |
|  | 1. Publication year |
|  | 1. Corresponding author region: continent |
|  | 1. Title |
|  | 1. Funding sources reported:  - yes (no conflict) - yes (conflict unclear) - no |
| **Study design** | |
|  | 1. Study type:  - primary study - review |
|  | 1. Review design:  - systematic - other (rapid, scoping, overview of reviews) |
|  | 1. Review publication date |
|  | 1. Review primary studies: number of articles |
|  | 1. Review published primary studies: number of articles |
|  | 1. Review unique published primary studies included: number of articles |
|  | 1. Review published primary studies not identified in our search: number of articles |
|  | 1. Primary study design:  - randomized - nonrandomized (eg, questionnaire, interview, focus group) - both (if review) |
|  | 1. Primary study data:  - quantitative - qualitative - mixed |
| **Study aim and focus** | |
|  | 1. Study **aim** from abstract |
|  | 1. Study **focus**:  - Effectiveness (or efficacy) - feasibility (eg, acceptance, engagement, usability, or adherence) - (program) evaluation |
| **Population (older people)** | |
|  | 1. Sample size: n participants per primary study or in all studies in review |
|  | 1. Age:  - Cut-off age (eg, 60+) - range or mean range, mean±SD, median |
|  | 1. Gender:  - any, male, female - % female |
|  | 1. Health status:  - any, healthy, at risk (for any disease), clinical (with any disease) - diagnosis (risk for or existing disease) |
|  | 1. Country of primary data collection |
|  | 1. Employment status:  - employed (some or all) - not employed (all or majority) |
|  | 1. Socioeconomic status of all or majority of participants (estimated from income or other indicators: high or low) |
|  | 1. Education level of all or majority of participants (estimated from completed school level: high or low) |
|  | 1. Digital health competence estimated based on experience and use of digital technologies (high or low) |
| **Concept (digital technology)** | |
|  | 1. Digital technology **type**:  - any - internet - telehealth - eHealth - mHealth - virtual reality - mobile technology - other (example) |
|  | 1. Digital technology **device**:  - computer (desktop, laptop) - mobile phone - smartphone - tablet - wearable - gaming console - other (example) |
|  | 1. Digital technology device **use**:  - website - text-message - email - app - exergaming - other (example) |
| **Context (health promotion and disease prevention)** | |
|  | 1. Health target **category**:  - mobility - nutrition - cognition - mental health - other (eg, sleep) |
|  | 1. Health target category **parameter** according to study authors:  - mobility: eg, physical activity, balance, falls prevention, gait, muscle strength, coordination - nutrition: eg, diet, weight management, BMI management, blood sugar management - cognition: eg, executive functioning, motivation - mental health: well-being, quality of life, mood and stress management, social functioning, substance use management - other (eg, sleep, general healthy aging) |
|  | 1. Health **purpose** (can be estimated based on items 26-28):  - feedback on performance - engagement (eg, logging into website) - monitoring or tracking of own health - reminders - recommendations or advice - education - goal setting - social networking or support - other (eg, rewards, motivation) |
|  | 1. **Setting**: any non-clinical (eg, daily life, home, community), or other (example). Note: if NR explain why setting correct (eg, healthy participants and exclusion criteria hospital, care home, rehabilitation facility) |
| **Digital technology use pattern** | |
|  | 1. Duration: in months, mean or range |
|  | 1. Attrition: % of total study sample |
|  | 1. Use benefits and opportunities based on study results, discussion or conclusion: eg, acceptability, engagement, health target improvement, or other (example). Note: consider if health target improved compared to baseline, other active control condition (eg, other intervention) or inactive control condition. |
|  | 1. Use challenges based on study results, discussion or conclusion: eg, reasons for attrition, difficulties with use, suggestions on how to improve use or other (example) |
| **Evidence gaps** | |
|  | 1. Study conclusions from abstract |
|  | 1. Ideas for future research from study discussion or conclusion |

Note. If information is missing in studies insert NA = not applicable or NR = not reported.

## Textbox S2. Results in 90 primary studies.

### Bibliographic Characteristics of Included Studies


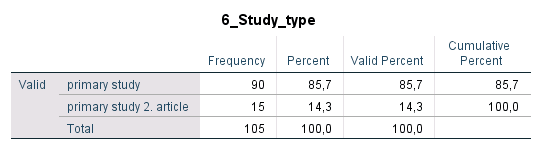


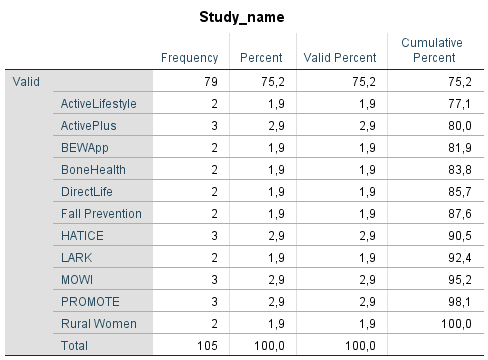


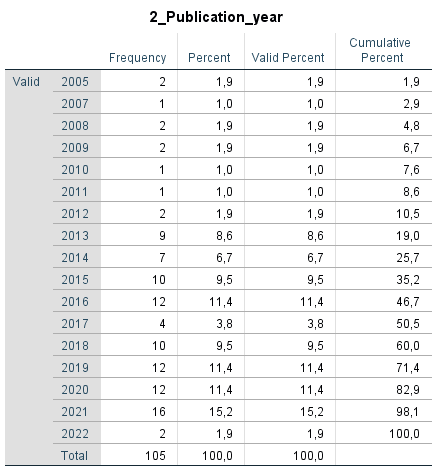


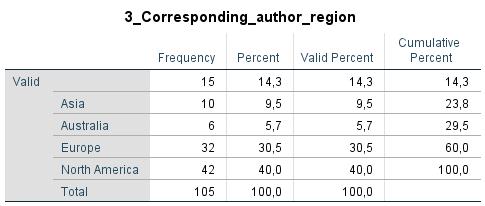


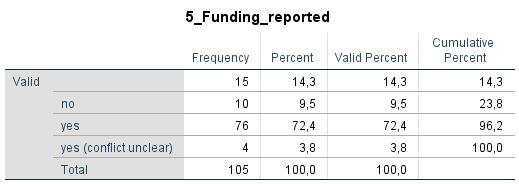


### Objective 1 (Studies): Study Designs and Aims


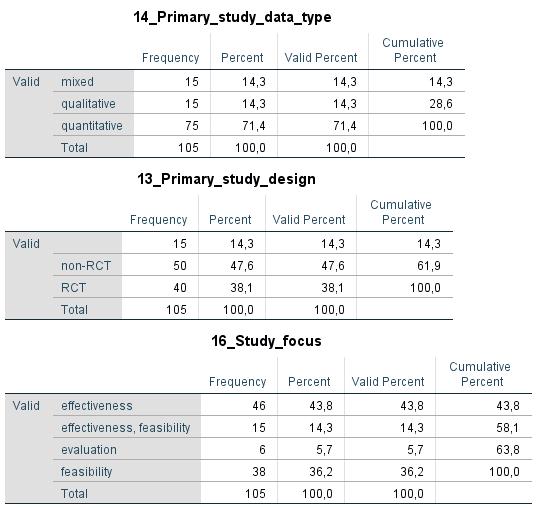


### Objective 2 (Population): Older People as Users of Digital Technologies


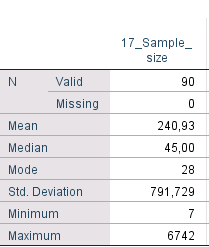


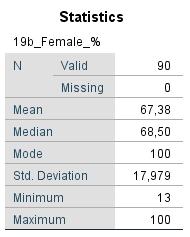


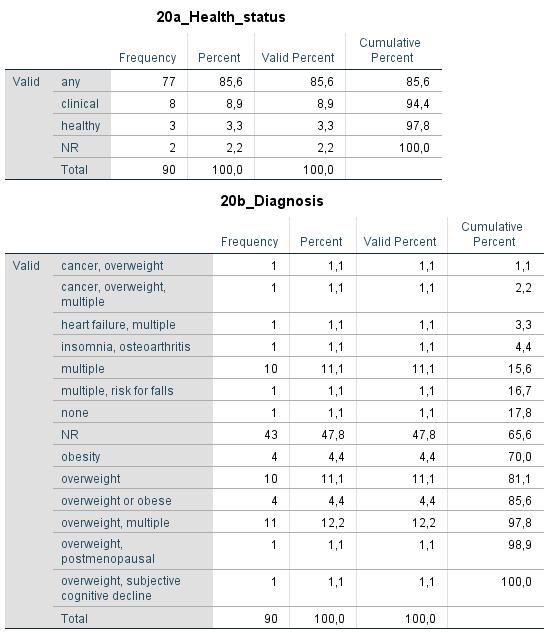


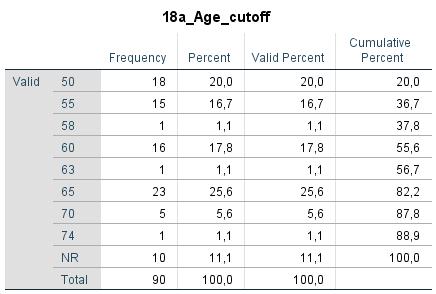


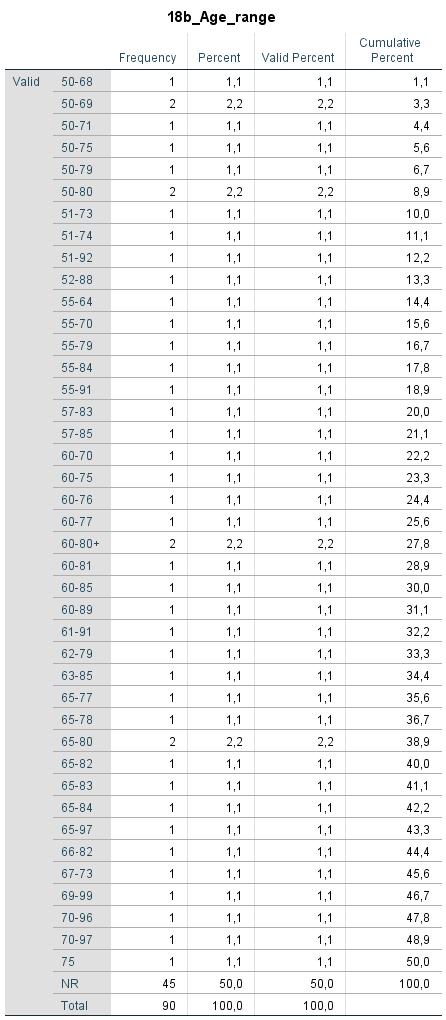


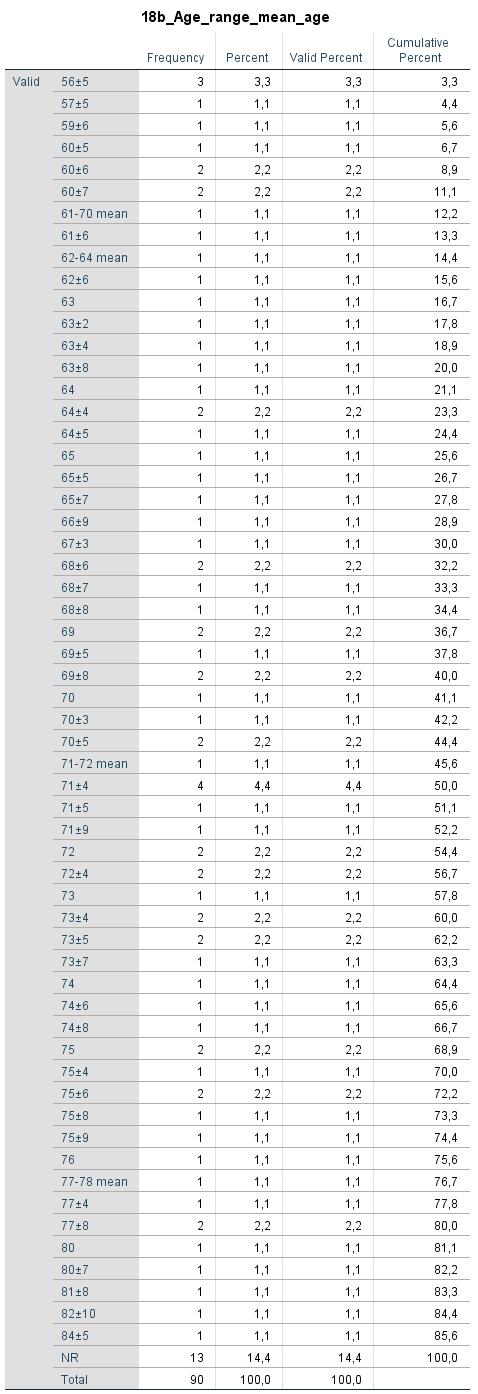


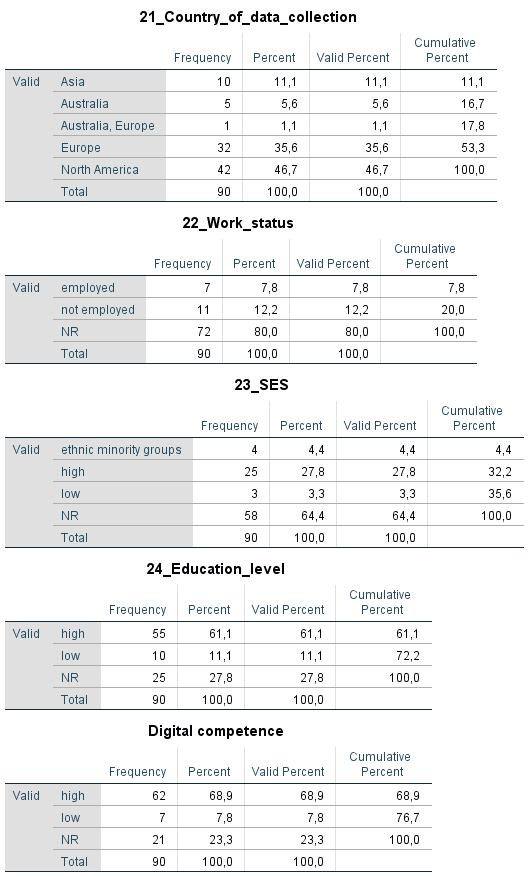


### Objective 3 (Concept): Digital Technologies Used by Older People


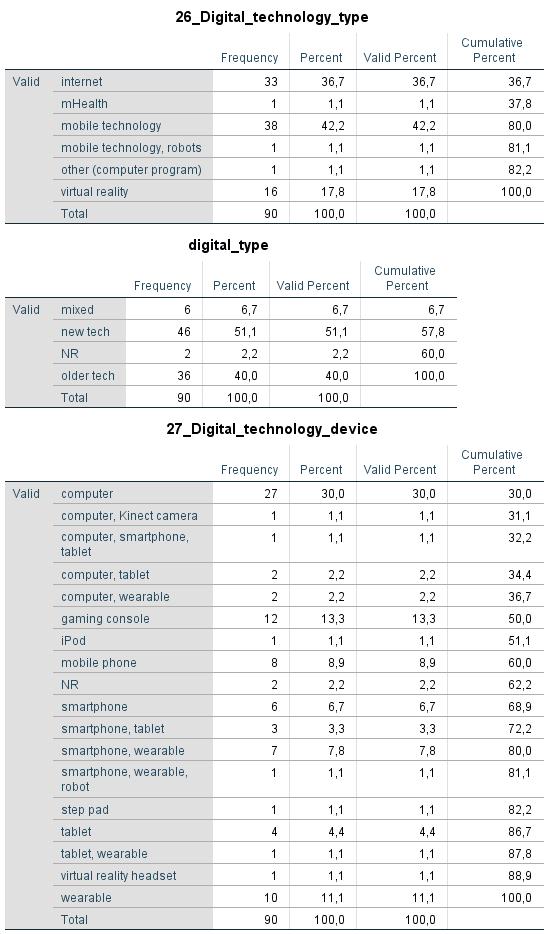


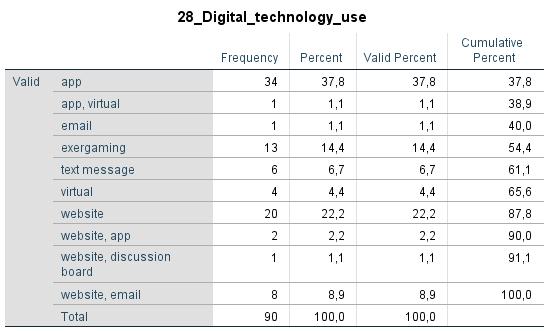


### Objective 4 (Context): Health Targets of Digital Technologies


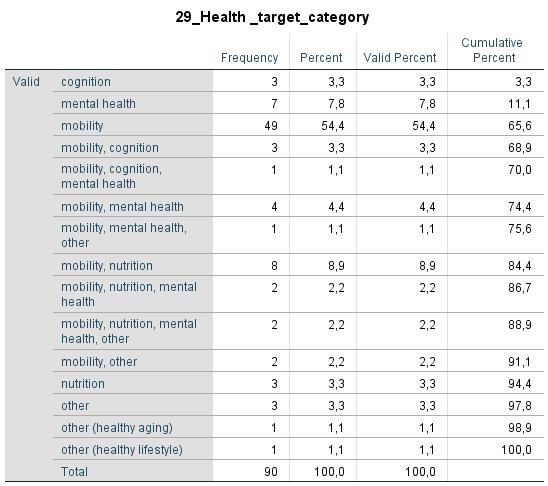


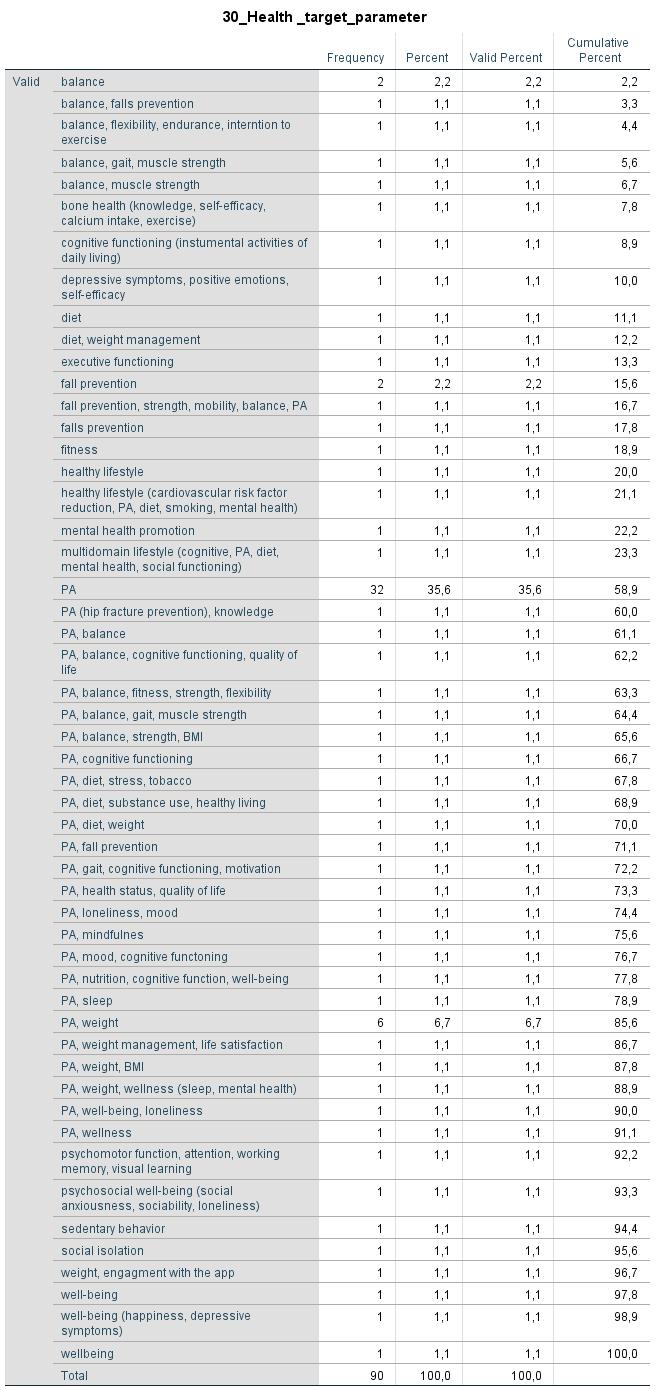


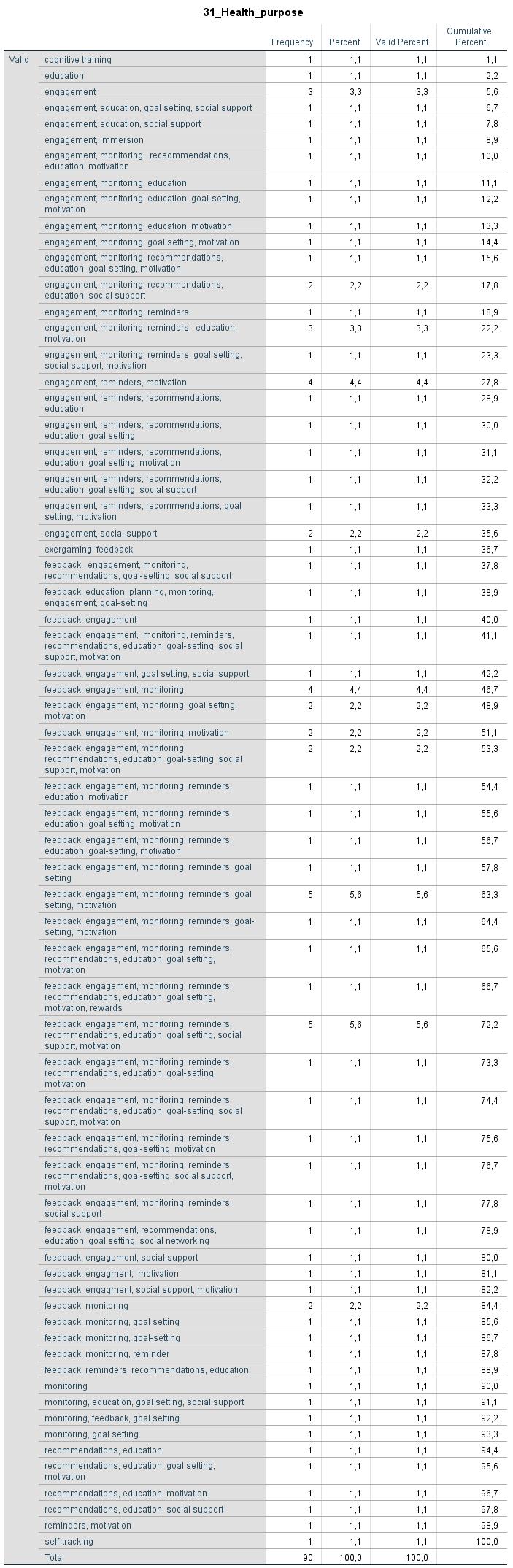


### Objective 5 (Use Pattern): Opportunities and Challenges with Digital Technologies


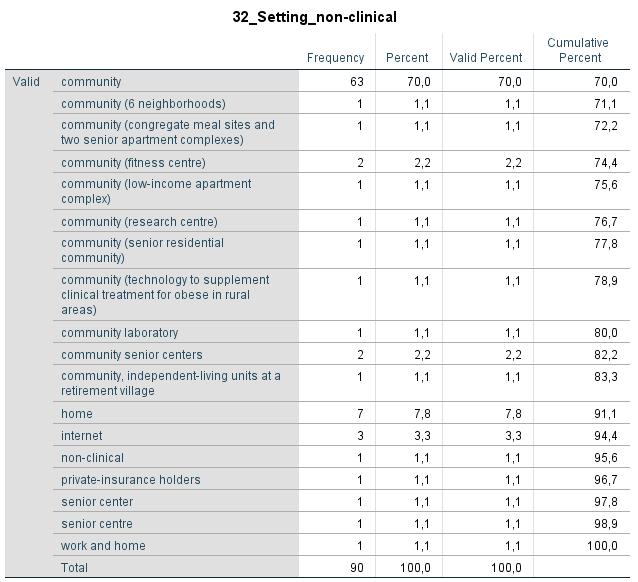


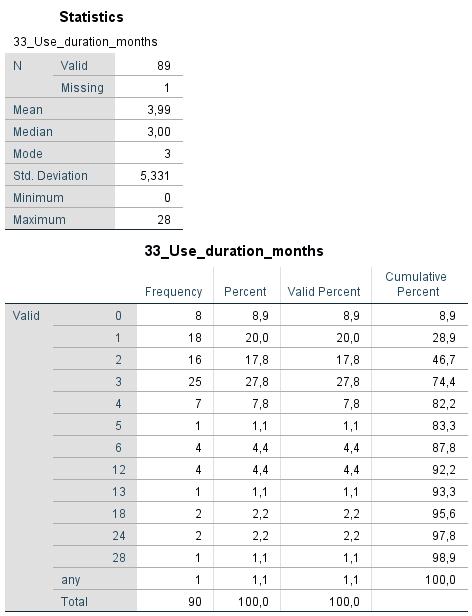


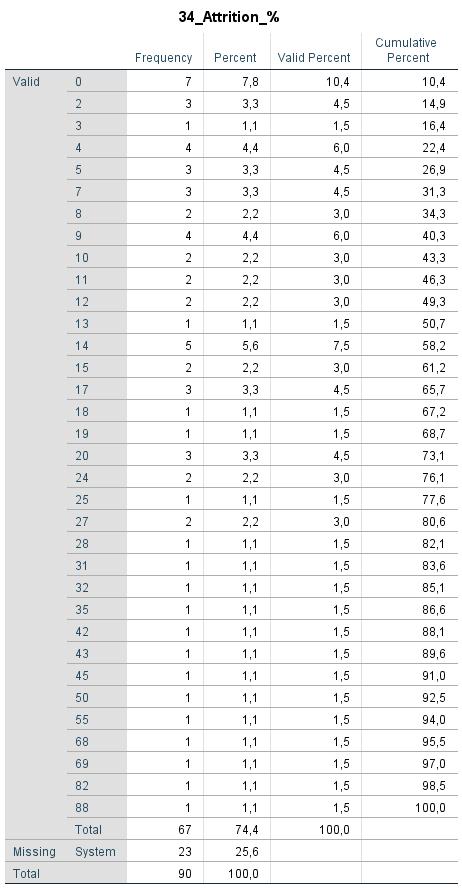


**Opportunities**

| 1 | ↑ PA, ↔ cognition vs. control (printed material); high usability, acceptability |
| --- | --- |
| 2 | PA ↔ tailored vs. standard newsletter |
| 3 | ↑ Intention to carry out exercises tailored vs. non-tailored website |
| 4 | ↑ weight loss website+peer vs. website only, internet delivery feasible |
| 5 | ↑ computer operating skillls, health-related knowledge, frequency of performing exercise vs. baseline |
| 6 | ↑ satisfaction and enjoyment (sharing health behavior and learning from others) |
| 7 | ↔ self-efficacy, knowledge, ↑ satisfaction, exercise website+discussion vs. website only |
| 8 | 65 years or older: ↑ engagement with the program (high frequency of recordings of food intake and current weight), women ↑ weight loss, men ↑ engagement (logins) with the website vs. Younger than 65 |
| 9 | ↑ trends in less loneliness and improved mood in Wii vs. TV watching or control |
| 10 | ↑ PA, time spent on website vs. younger age groups; moderate acceptability, high usability of website |
| 11 | PA, weight loss, BMI reduction, psychometric measures (motivation, goal setting) ↑ walking program vs. baseline; ↔ human coach vs. no coach |
| 12 | ↑ physical outcomes vs. control (no online intervention) and baseline |
| 13 | ↑ step count, leisure time exercise vs. control (no text-message) and baseline |
| 14 | ↑ PA, motivation vs. inactive control and baseline |
| 15 | ↑ mood, ↔ cognitive function vs. baseline, enjoyable and fun activity |
| 16 | ↑ PA vs. baseline and control (no monetary reward) |
| 17 | In-depth appreciation (e.g. reliability, perceived individualization) ↔ vs. control (print); use and appreciation of information ↓ vs. control (print) |
| 18 | PA at 12 month follow-up ↔ vs. control (waitlist), ↓ vs. control (print) |
| 19 | PA ↔ vs. control (print) and ↑ vs. baseline, sufficient PA days ↓vs. control(print) |
| 20 | ↑ PA, social motivation more effective vs. individual motivation to perform exercise; app assisted and motivated to autonomously perform exercise; high engagement with app |
| 21 | ↑ PA in tablet vs. paper brochure groups |
| 22 | PA, body composition ↑ vs. control (waitlist) |
| 23 | overall health and quality of life ↑ vs. control (waitlist) |
| 24 | high acceptability, good usability among participants who tried the app for 7 days (app learnable, satisfying, effective) |
| 25 | ↑ awareness of diet monitoring, social support via discussion board |
| 26 | weight loss, healthy diet ↑ vs. baseline or ↔ vs. control (email intervention) |
| 27 | well-being outcomes trend ↑vs. baseline, ↔ vs. control (placebo intervention online) |
| 28 | ↑ muscle strength, gait, ↔ balance vs. baseline |
| 29 | ↑ PA, perceived health vs. baseline; liked physical and social components of exergaming |
| 30 | ↑ PA in tracker group vs. control; high tracker use on 95% of intervention days; 96% reported liking the website and 100% liked the tracker; high engagement with the tracker (72% viewing tracker data ≥1 time/day), a passive approach to the website (viewing feedback but not changing goals or manually logging behaviors). |
| 31 | PA, diet, self-efficacy, exercise planing, aging beliefs ↑ vs. control (waiting list) |
| 32 | reasoning ↑ in all or subgroup at risk for cognitive impairment vs. control or baseline |
| 33 | ↑ PA, cognitive functioning, ↔ quality of life in exergaming+educational information vs. control (educational information), high enjoyment, usability |
| 34 | ↑ health behavior or intention, usefulness, easiness vs. baseline |
| 35 | ↑ PA level and awareness, satisfaction with intervention: mixed vs. control (no intervention), ↑ PA awareness website vs. control |
| 36 | walking parameters ↑ vs. baseline and control (no intervention) |
| 37 | ↑ PA vs. baseline, regular engagement with wearable (downloading data at least once a week) |
| 38 | iPad apps could help older people monitor and improve their diet (locate inexpensive food sources, obtain recipe recommendations and communicate the diet in clinical context) |
| 39 | exercise intention, social presence ↓ exergaming vs. control (traditional exercise) |
| 40 | ↑ PA vs. control (no text message) |
| 41 | ↔ PA, ↓ exercise confidence vs. baseline; high satisfaction with device, easy to use, feedback useful |
| 42 | ↑ healthy living behaviour and intention SMS vs. baseline, ↔ SMS vs. control (classroom training) |
| 43 | subthreshold despression, positive emotions, self-efficacy ↑ vs. baseline and control (low dose intervention) |
| 44 | wearable was easy to use, useful, and acceptable at 10 weeks (with social support) and 8 months follow-up (without support), ratings dropped from 10 weeks to 8 months |
| 45 | PA intervention supported by app ↑ PA behavior, balance, intention to exercise vs. control (motivational content without app), high acceptability of PA intervention |
| 46 | PA (exercise frequency) at 12 weeks ↑ and at 24 weeks ↔ vs. control (no text-messages), text messages useful at improving motivation |
| 47 | program feasible at continuous monitoring of cognitive functioning over time |
| 48 | PA in different domains (walking, gardening) ↑ vs. baseline and vs. control (waiting list) |
| 49 | psychosocial wellbeing ↑ vs. baseline in groups playing with others (younger or older people) |
| 50 | high satisfaction and acceptance, ↓ adherence over time, ↔ health status, quality of life |
| 51 | trend ↑ PA, weight loss digital intervention+coaching vs. control (coaching), high acceptability, satisfaction and usability |
| 52 | bone health outcomes ↑ vs. control (no intervention) or baseline |
| 53 | balance ↑ vs. control (cognitive online intervention) |
| 54 | PA ↑ vs. inactive control (logbook), ↓ satisfaction, ↑ time spent on the website in older vs. younger than 55; older: overall ↓ satisfaction, usability, interaction with website over time (0 to 18 months) |
| 55 | Usability can be improved if wearable device is comfortable, can communicate with smartphone or tablet and has large screen. Measurement accuracy and human support are needed for effectivness. |
| 56 | digital technology could help tracking health, improve patient knowledge (insight into health), motivating change and assuring accountability |
| 57 | Digital technology has potential to  improve health and create accountability. Technology might overcome geographic barriers to connect people in rural areas and allow to deliver intervention at home to reduce embarrassment about weight |
| 58 | ↑ acceptability (comfortable with session format, instructions clear), social support, ↓ isolation |
| 59 | PA outcomes, life satisfaction ↑ vs. baseline in prefrail group, better outcomes during coaching vs. self-management phases and in people wearing mobile devices for the longest period |
| 60 | ↑ cognitive functioning and mental health (↓ anxiety, depression) vs. baseline; high engagement, compliance and satisfaction with the program |
| 61 | PA ↑ vs. inactive control (no text message with PA content) |
| 62 | heterogeneous acceptance of intervention, ↑ usefulness and mindfulness, low usability without digital competency |
| 63 | older adults are open to using technology-based exercise programs in their home; program uptake and adherence depend on perceived benefits (enhancing motivation), whether the program was stimulating and easy to use |
| 64 | factors necessary for sustained engagement: human support, automatic and personal reminders and tailoring, clear expectations of the platform, incorporation into daily routine |
| 65 | ↑ or trend improvement in some healthy lifestyle outcomes (reduction in cardiovascular risk factors, PA, diet, les smoking, less anxiety) vs. control (website without human coach) |
| 66 | higher engagement was associated with significantly greater improvement in biological and behavioral risk factors, with evidence of a dose-response effect (change in the primary outcome ↑ in the high and moderate engagement groups vs control group), any outcome measures ↔ low engagers vs. control group |
| 67 | telehealth physical therapy-led intervention to increase physical activity in low-income older adults aging in place was successfully implemented and attended |
| 68 | high motivation to use technology for mental health promotion |
| 69 | Attitudes toward using technology in daily life only changed in the physical domain, but noticeably, with all participants perceiving an added value after use |
| 70 | Some PA outcomes ↑ vs. Baseline, high acceptability and usabilty, enjoyment |
| 71 | high satisfaction; fun and easy to use, no help needed for easy tasks |
| 72 | PA, sedentary time ↔ vs. inactive control (waiting list); PA ↑, sedentary time ↓ in website+tracker vs. Baseline, but not in website alone |
| 73 | effects of web-based intervention on PA depend on socio-cognitive factors (task self-efficacy, intention, and action planning) |
| 74 | different acceptability and requirements by men vs. women, younger (<70 years) vs. older (≥70 years)- perceived enjoyment and benefits of socializing while exercising together, the time expenditure perceived to be acceptable, previous digital skills, perceptions that ambience and accessibility of exercise facilities in the neighborhood were important. |
| 75 | Fitness apps used most frequently for PA, diet and weight management, heart monitoring, relaxation |
| 76 | no outcomes measured, attitudes towards fitness apps |
| 77 | multidomain intervention (in person and computerized) improved or maintained cognitive functioning in at-risk elderly people from the general population |
| 78 | ↑ some PA outcomes in daily living vs. control and baseline |
| 79 | ↑ sleep, ↔ PA vs. Baseline |
| 80 | some ↑ cognitive functioning, perceived health, PA ↔ vs. inactive control |
| 81 | PA outcomes ↔ vs. Baseline |
| 82 | Older people (>65) vs. <64: majority of apps downloaded by older people health related; use sustained for a longer time, 63% self-tracked health measures, but the majority did so manually |
| 83 | 43% chose digital program over paper booklet program; ↑ PA, satisfaction and support from program, maintenance at 12 months, lower attrition in digital vs. paper program |
| 84 | digital program perceived as a supportive to guide training and tailor the program; supporting autonomy to exercise considering difficulties with perception of exercise in older age; useful guideance via instruction videos |
| 85 | intention to use predicted by perceived usefulness, enjoyment, ease of use |
| 86 | balance trend ↑ vs. Baseline, high acceptability and adherence (sense of achievement, guidance, clear instructions) |
| 87 | Sensor-controlled digital games (SCDHs) that can integrate real-time behavior data from behavior-tracking sensors into mobile game acceptable; game can encourage self-management in real-life; 90% recommend the game, 100% felt motivated |
| 88 | aerobic PA, motivation to exercise ↑ morning text message vs. control (text message without PA content) |
| 89 | fall-related parameters ↑ vs. baseline |
| 90 | weight ↓, PA ↑ vs. Baseline, high satisfaction with the wearable |
| 91 | weight ↓, PA ↑ vs. baseline, high satisfaction and usability of a wearable |
| 92 | PA ↔ vs. Control (waitlist or health coaching); high adherence and engagement but low use of PA aspects of digital technologies; ↑ awareness of sedentary time |
| 93 | PA, perception of own physical and mental health ↑ vs. baseline |
| 94 | fall outcomes ↔ vs. Control (health education without online program) at 12 months, ↑ at 24 months; high adherence |
| 95 | older adults 65+ ↑ engagement (more coaching conversations, logging more data and higher device logs) especially in the wellness context than younger people (35-64) |
| 96 | ↑ weight loss associated with ↑ engagement vs. baseline |
| 97 | no effects of smartphone app vs. control (education program) on health outcomes |
| 98 | participants believed digital tools could positively contribute to improving their overall wellbeing; usability depends on user-friendliness, facilitating communication, improving empowerment (prolonging independent living, promoting freedom) and quality of care at times of increased demand for care |
| 99 | PA ↑ fall rate ↓ vs. baseline |
| 100 | ↔ PA, ↑ computer time vs. Baseline |
| 101 | ↑ PA active users (more than 30 day of use) vs. less active or non-users; 71% used wearables for 3 or more days; wearable use associated with better cognitive functioning; reduction in wearable use over time (baseline to 12 months) |
| 102 | experimental condition ↑ PA, social engagement app+social contacts vs. baseline; ↔ PA in app only (no social component) vs. baseline |
| 103 | heart rate monitoring, ↑ PA vs. Baseline, high usability and satisfaction |
| 104 | PA at high intensity ↑ audiobook+activity tracker vs. control (no audiobook), satisfaction with combining PA and audiobooks; high satisfaction and awareness of PA |
| 105 | Retention, engagement, satisfaction high and sustained, weight loss |

**Challenges**

| 1 | usability and acceptability high although technical problems reported (data entry, operating the touch screen), more realistic designs needed (less repetition, more possibilities for free expression), ethical issues (deception should be avioided by explaining that the agent is not a real person) |
| --- | --- |
| 2 | Human support helpful (retrieving the first newsletter); tailored advice helpful; digital divide (online interventions available for wealthier, highly educated) |
| 3 | tailored advice helpful; knowledge of internet |
| 4 | peer-support important to access intervention, some initial technical support required, multimodal engagement with the intervention important (website logs and e-newsletter) |
| 5 | computer language difficult, limited finacial resources (no personal computers), lack of computer support, poor eye-hand coordination, back pain when sitting |
| 6 | Online discussion boards require adequate computer skills, improve motivation to change behavior and improve learning about health |
| 7 | effectivness of online intervention (learning modules) depends on health competence, links to other health websites could provide additional learning benefits, websites developed specifically for older adults, help desk needed if new modes of online communiation (discussion boards) used, internet use should be encouraged to disseminate health information to older adults |
| 8 | Unclear how the older participants interacted with the website in this real-world program not designed as a scientific study |
| 9 | social aspects of playing Wii with another person can improve loneliness more than watching TV with another person, complex relationship between loneliness, positive mood and PA; Wii provides opportunities to engage and psychologically benefit from previously enjoyed activities |
| 10 | no contact with study participants is required to mimic the real life conditions of using internet independently; tailored advice is based on typically overestimated self-reported PA-levels and thus has low acceptability in any age group; websites are effective if they include little text and links, big buttons and fonts to be more user friendly for older people; acceptability of the website was similar in all age groups despite lower internet confidence in older people |
| 11 | digital intervention should not interfere with daily life (eg, work); high attrition if too complex (reliance on collecting and entering own data); continuous technical support needed; tailored intervention might evoke better psychological responses |
| 12 | self-tailoring effective (can be easily done digitally), educational content important for user satisfaction (easy language, easy content with examples in different modalities- videos and text), reminders important to improve compliance, effectivness depends on the dose (greater use leads to greater outcomes), potential for fraudent information in fully-digital interventions with rewards (young people signing up for profit) |
| 13 | text-messaging can be effective also in low SES populations, health communication interventions need to be based on behavioral theory to be effective |
| 14 | effectiness depends on cultural adaptation and language, use of simple technology that requires minimal digital literacy and health literacy, simple devices that can store and transfer data to a computer required for low SES people with low numeracy, interventions for older people may be effective even if they promote low intensity exercise |
| 15 | access to equipment (Wii), even single session of a digital intervention can have short-term benefits on mood, digital interventions can be feasible for home-bound people with poor physical health, some concerns about safety (eg, falling off the equipment), motivation to continue training, unclear outcomes for individuals although feasible for the age group, unclear if feasible for inactive, frailer people |
| 16 | additional rewards (monetary) might be required to improve effectivness and motivation to use digital tools for physical activity; location as a barrier to treatment (internet removes the need to travel to participate in intervention) |
| 17 | different mechanims of information processing depending on intervention demands (eg, printed materials were used more frequently but internet version provided immediate feedback on goals); difficulty using computers; environmental components (eg, information on local cycling routes) were used more than a simple intervention (digital or print without additional environmental information) |
| 18 | more planning required (computer needed), website updates and better appreication and usability of online materials required to maintain participant interest, effectivness depends on exercise intention and engagement with website; outcome measures need to be measured with validated methods instead of self-reports |
| 19 | distraction due to hyperlinks, too much information (overload), low familiarity with the internet, effectivness of internet better in younger 50-64 than older 65+ participants, effectivness depends on outcome measures (eg, active days/week or minutes of physical activity per week) and participant characteristics (eg, younger participants may still work and thus have less physcially active days than retired participants) |
| 20 | Metaphors to growing plants in a garden useful to motivate promoting and development of PA level; large buttons and fonts; using tablet+social group was more motivational vs. tablet or paper booklet alone; lower attrition in tablet+social group than individual exercises; intervention imporoved digital competence in participants (bough own tablets, started using the internet socially) |
| 21 | social support from coaches required, higher PA benefits in active vs. inactive participants; motivational aspects, an attractive design, automatized reminders, and the opportunity to give feedback about performed exercises to training supervisors seem to enhance adherence and compliance rates |
| 22 | objective measurements (eg, accelerometer) required to assess outcomes, position of the tool can affect results (eg, better on the ankle vs. wrist), stimulating behavior change requires personalized feedback, providing accelerometers to controls also contributed to improved outcomes; reduction in face-to-face data collection can contribute to higher adherence to intervention; effectivness probably depends on high motivation; outcome assessment can be blinded when using objective tool data |
| 23 | improved PA outcomes are associated with improved quality of life, especially emotional aspects in inactive adults (dose-response relationship); improved quality of life depends on social support and tailoring of intervention; self-report and accelerometer data (quality may report on the location of the sensor, eg on one side of the body or on wrist vs. ankle) |
| 24 | clarity of app content (eg, motivation content) needed, additional information needed on charging the device and activating the app, user-friendly images needed (eg, a garden), mobile device not always appropriate to wear, human support was needed to fix any technical problems, difficulties with tapping the monitor |
| 25 | website content incomplete (not tailored towards individual eating habits), environmental factors (eg, restaurants) not in the area |
| 26 | computer training required beforehand, easy access to the internet can help to track dietary behavior in real time, internet and emails were equally effective (tailoring of delivery may further improve effectiveness) |
| 27 | intervention used by younger people; more support than emails only may be required to improve adherence |
| 28 | video games can increase motivation to engage in exercise; designs of exergames needs to consider effects but also risks (eg, falls); highest benefits if exercise guided by physiotherapist |
| 29 | technical issues (time to set up the equipment), finding appropriate location for exergaming; human trainers necessary to support the training, real-world settings necessary but convenience samples may be more motivated to participate in physical exercise |
| 30 | no technical issues in people with high digital competence; interactive technologies more useful than traditional pedometers: 96% rated the Fitbit as “somewhat or very” helpful for increasing PA, compared to only 32% of the Pedometer Group who found the basic pedometer “somewhat or very” helpful; low motivation to explore the website beyond its basic functions |
| 31 | outcome changes with small effect sizes; program effective especially for women and especially for planning but not performing exercise; technical support provided; self-reported data; potential ceiling effect (program attracted well-educated and affluent people with good health practices at baseline) |
| 32 | high attritition if engagement is not encouraged and no support is provided |
| 33 | exergaming feasible for independent training at home, but adherence (minutes/week) low |
| 34 | limited availability of equipment, training possible only at a senior centre, too little training (twice a week) to elicit PA changes |
| 35 | support needed with computer and website accessibility, effectivness depends on social interactions and tailoring of intervention (eg, adjusting PA levels and providing tailored recommendations online) |
| 36 | unclear if older people can operate the equipment independently; feedback from the gaming console can further improve outcomes |
| 37 | high intervention uptake in participants with high digital competence |
| 38 | limited knowledge on iPad’s functions; high costs of iPads, practice and instruction necessary to overcome technical problems; iPads more useful than computers (portable, touch screen easier than a mouse) |
| 39 | preferences of older adults and customized elements required in development of exergaming for the elderly; social interaction necessary for effectiveness |
| 40 | PA increased in a real-world setting but with fit and affluent participants; working older adults have less time to participate in health interventions |
| 41 | benefit of self-monitoring and participant motivation, but social support important to improve confidence |
| 42 | Characteristics of text messages can influence the effectiveness (frequency, style, time of day when the message is sent). |
| 43 | high motivation may further enhance the positive effects of exergaming; effectivness can differ depending on exercise form and length |
| 44 | without social support acceptability and usability of wearables decreases, especially among the oldest (80 or older), goal setting in commercial devices (eg, FitBit) need to be adjusted for older adults (eg, daily goal of 10000 steps may need to be reduced for sedentary adults), importance of social learning of new technology with other people than alone, ownership of a wearable unlikely to elicit behavior change (engagement and human support required) |
| 45 | PA interventions may be more effective (more motivational) when supported by wearable devices, wearable devices useful at recording PA data |
| 46 | text messages useful only in those with low motivation to exercise, frequency of text messages important (ignored if too often), tailoring of frequency needed (more motivated need less text messages), human support need but also costly to implement and could reduce outreach in interventions |
| 47 | support needs to be provided to resolve technical difficulties with compupter and website use |
| 48 | eHealth intervention feasible in adults with low education, factors that contribute to effectiveness of eHealth interventions: informal intervention, use of individual tailoring, goal-setting and monitoring, targeting daily life activities, being low cost |
| 49 | effectivness of exergaming highest when games are played with others and not alone |
| 50 | technical issues (too slow internet, lack of program updates); technial support provided, social support and supervision could improve adherence, effectiveness might depend on exercises the participant likes, time of the day, age, and gender |
| 51 | equipment damage or loss (tablet, wearable); social interactions among participants via app and professional coaching contribute to effectivness of any intervention |
| 52 | internet can facilitate knowledge dissemination to improve health behavior |
| 53 | effectiveness of exergaming is counfounded by social contact with research staff and depends on a dose, self-guiding and tailoring improved adherence, |
| 54 | internet self-efficacy positively associated with usability and satisfaction with the intervention; acceptability of the intervention depends on how well the internet is integrated in daily lives of older adults |
| 55 | Effectivness and use can be reduced in people with cognitive decline. |
| 56 | access to digital technology difficult (no internet), complexity, personalization of messages required, privacy concerns |
| 57 | More social connectedness desired. Videoconferencing suitable to deliver interventions for geographically isolated people |
| 58 | lack of access to hardware, low computer competency, more social interaction desired |
| 59 | outcomes depend on compliance with wearable device, social support and reminders needed to improve compliance |
| 60 | coaching required for better outcomes |
| 61 | the message content and time (morning) is effective at prompting PA in physically active people; text messages with mental imagery may be an easy-to-use, cost-effective and beneficial method of motivating PA in older adults |
| 62 | interface and content not targeted for older adults, technical issues decreased adoption of intervention, poor attitudes to technology associated with lower use of intervention, intervention was possible only after learning to operate the touch screen and activating the intervention, applicable for real life intervention (practical exercises) |
| 63 | digital interventions must be beneficial at improving health outcomes, stimulating physically and mentally, accessible at home and designed for older adults (program design and usability); difficulty using the system |
| 64 | use depends on perceived computer literacy |
| 65 | intervention could be used for broad, low-cost implementation in settings, including low-income and middle-income countries, where internet access is rapidly increasing; confounding of the effects of digital intervention by human coaching; intervention designed with and for older people |
| 66 | health inequalities (those with poor digital literacy, cognition, education engaged less) |
| 67 | adherence to exercise, but low use of wearables at 1 year follow-up; recharging the wearable (had to remember to do); video-based exercise instruction acceptable; high preference for exercising in a group setting to reduce social isolation and depression |
| 68 | technology is inferior to humans, but can promote self-reliance among older people who want to reduce the burden of their low mood on others; low readiness to use technology with low mood, lack of technology knowledge (how to use some features of apps); awareness of digital technologies did not motivate their use; apps and websites should use intuitive layouts with easy-to-use function buttons and clear explanation; physical problems (poor eyesight, dexterity) with operating and understanding the technology |
| 69 | little awareness of health advantages of technology; technology acceptable for PA and weight monitoring, well-being was not perceived as a health domain or it was not clear how technology can be of any support; fears from technology mentioned were attention theft, replacement of human touch, and disuse of existing abilities; short period of use contributed to better attitudes towards technology; training of cognitive function acceoptable but not monitoring or feedback if results suggest cognitive decline over time; keeping a log of food intake not practical (takes too much time); app content related to daily life too repetitive |
| 70 | little technical difficulties, social component of intervention important to motivate participation in a group PA |
| 71 | complex tasks within an app may be difficult to achieve (eg, setting up own exercise schedule); privacy (entering own data) problematic, more instruction needed |
| 72 | unclear if effectiveness depends on digital technologies alone or an add-on effects of group exercise |
| 73 | self-efficacy and planning should be adressed for effectiveness |
| 74 | Digital dairy easily integrated in daily life, but time consuming; technical problems with wearable use and data storage and transfer; website should be more tailored; technical support and training in website use necessary |
| 75 | low interest in apps (even if the app were recommended based on scientific evaluation), worried about data protection, apps too commercial or not serious; 60% use apps regularly; highest interest in apps in physically active people; helpful PA apps features: setting and feedback on behavioral goals, instruction on how to perform behavior |
| 76 | Fitness apps should be easy to use with few features and include automated tracking rather than manual data entry; feedback, self-monitoring and social comparisons helpful to reach own PA goals; concerns about data safety; heterogeneous appreciation of different feastures in apps |
| 77 | low preference for computerized cognitive training vs. in-person intervention components, only 12% completed all training sessions: previous experience with computers, better memory performance, and positive expectations toward the study predicted starting the program, previous computer use associated with a greater number of training sessions completed |
| 78 | increasing light-intensity mobility may be already beneficial especially to perform the activities of daily living (walking, gardening), planned behavior may not always be executed (objective measures of PA are needed), telephone reminders might help to limit dropout, but may not be feasible in larger studies |
| 79 | high fitbit wear time without reminders, technical support provided |
| 80 | intervention performed in goups, social contacts confounding the results |
| 81 | smartphone app easy to use, clear design, attaching and wearing the self-monitoring device problematic, frequency of consulting the app highly variable (0- 20 times a day), motivating, surprising, and interesting, became more aware of their sedentary behavior but did not change actual behavior |
| 82 | high satisfaction with mHealth, no difference to general population; intended use depends on ease of use, satisfaction and perceived usefulness; once using smartphones older adults do not differ from younger (eg, use multiple health apps); integration of app data into healthcare system |
| 83 | collecting data on program adherence is complex (unclear how participants interact with the intervention), different  ways of self-reporting exercise |
| 84 | autonomy depended on participants’ competence in exercise and the use of technology; social support needed; group-based exercise requested |
| 85 | less self-efficacy (less confidence in own PA) and higher need to stay fit lead to more perceived usefulness |
| 86 | lack of variety, not enough progression, need to please the researchers |
| 87 | technical issues of navigating though the game, educational information too trivial for high SES participants |
| 88 | text messages contributed to effectiveness of a mindfulness intervention on improving motivation to exercise but only when sent in the morning |
| 89 | high engagement, easy to use independently, high level of engagment due to feedback from avatar, personal coaching and technical support, technical problems when logging into the app, challenging program with increasing PA demands enjoyed by the participants |
| 90 | internet access and technical skills to synchronize Fitbit device data remotely; effectiveness could improve with video-conferencing component |
| 91 | Fitbit integrated among a multicomponent intervention in older adults, unclear whether the use of technology  enhanced the retention or effectiveness outcomes |
| 92 | coaching and technical support required, feedback (vibration) from the wearable ignored |
| 93 | difficult to replicate social interactions onsite in an online program, coaching and telephone support provided, social interactions encouraged in zoom |
| 94 | intention to exercise declined over time despite social support |
| 95 | declining physical and cognitive functioning that affects operating of digital devices (eg, interpret text, use touch-based interactions, natigate the app menu, take measurements with smart devices, connect devices and transfer data, and maintain battery charge); low trust in technology (privacy risks due to insufficient training); less opportunities to obtain access from healthcare providers than younger people; shortage of healthcare providers who offer digital healthcare; younger older adults |
| 96 | effectivness improved by multiple modes of engagement (digital with the app and non-digital behavior-based, eg, measuring own weigh), data from real-world-users needed in digital research (research study participants may not be representative) |
| 97 | app was user-friendly, but should be tailored (eg, intensity of exercise); participants with high motivation and high PA level at baseline, app use feasible for PA |
| 98 | safety concerns and ethical issues related to privacy, lack of human contact, risk of infantization by robots |
| 99 | difficulties in navigating the program (eg, completing surveys using external links), program offers a chance to exercise for people who cannot attend exercise onsite, potential of remote delivery to reduce costs and improve health impact in aging society, lack of social support |
| 100 | adherence to intervetnion (more computer time), chances to reach isolated populations (COVID-19, remote living) |
| 101 | potential difficulties in sustaining benefits from wearables, reminders or motivation programs needed to enhance adherence; using wearable may not change behavior |
| 102 | younger and educated older adults report the same digital technology use as younger people; simple technologies, auch as smartphone useful for older adults; effectivness depends on social support and if the devices are worn or carried by the participants |
| 103 | privacy concerns if commercial devices used, device accuracy difficult to establish for commercial products, usability, and data access (what data is collected and how can it be used); device was difficult to strap (fine motor skills required), technical difficulties with starting the app, syncing and emailing the data, cognitive involvement to use the device (start and end session); commercial devices may be discontinued |
| 104 | high access to technology in this age group (smartphones), combination of audiobooks and PA effective |
| 105 | health recommendations and accountability (e.g., via a behavioral coach) may encourage motivation and accountability; appropriate app layout (large tap area and font) and content of exercises; socioeconomic, primary language, and race/ethnicity that could affect usability |

### Objective 6 (Evidence Gaps): Ideas for Future Research

| 1 | usability of the system in care settings |
| --- | --- |
| 2 | long-term effects |
| 3 | if intentions also cause behavior change, if online advice should be supplemented by other forms of support? |
| 4 | study with diverse samples, effectiveness of peer-support vs. email counseling? |
| 5 | continual and regular provision of ehealth programs for older people |
| 6 | long-term effects of websites with discussion |
| 7 | use of internet-based programs as cost-effective alternative for older adults to manage their weight at home |
| 8 | develop programs that enhance shared positive emotional experiences to improve life satisfaction in senior residential communities |
| 9 | effectiveness of real-life interventions vs. control groups using with higher doses of exercise in other older adult groups (less motivated to participate in studies), objective measures of outcomes needed |
| 10 | true control groups (no technical support), effectiveness of other methods than phonecalls (text messaging and internet) |
| 11 | factors to maintain engagement with PA websites over time, validation of self-report data, effectiveness of the program beyond younger older participants |
| 12 | larger samples, specifıc demographic characteristics that might affect  results, theory-based research needed, control groups with no intervention to reduce placebo |
| 13 | accelerometry to validate self-reported data for precise outcome measures, effects of delivery setting (community centre vs. own home) |
| 14 | long-term motivation to maintain training especially alone at home, RCTs to determine the effects of using the Wii vs. another activity (playing a computer game) as a repeated exposure on health and on programme adherence; heart rate intensity measured using indirect calorimetry; standerdazied sessions; including less active and less healthy participants |
| 15 | to design effective interventions for maintaining high activity levels and promoting long-term effects |
| 16 | cost-effectiveness of internet-based vs. printed interventions, long-term studies |
| 17 | long-term effectiveness and maintenance |
| 18 | long-term effectiveness; contribution of other factors (eg, possible dietary changes) to improved physical activity outcomes |
| 19 | targeting diverse older adults sample, evaluating theoretical fidelity of technology, estimation of energy expenditure using objective methods |
| 20 | application of internet programs for self-management of weight control to promote healthly living |
| 21 | factors required to improve adherence to intervention; testing the clinical effectiveness of the intervention in clinical samples |
| 22 | effectiveness in participants with low exercise ability and feasibility of training alone to test if videogames could contribute to continuous and frequent engagement in exercise |
| 23 | objective measurement of physical activity; explore participant satisfaction with other gaming consoles, encourage independent use of exergaming |
| 24 | long-term benefits |
| 25 | longer-term posttests, objective outcome measurement rather than self-report, effectiveness unclear in populations with lower education and affluence |
| 26 | long-term engagement and implementation; effectiveness in care context and in people at risk for cognitive impairment |
| 27 | factors that could improve adherence to independent exercise and exercise dose that could improve the quality of life |
| 28 | application in public spaces and clinical context |
| 29 | focus on disadvantaged seniors (less wealthy, less digitally competent), adopting a participant-centered approach, exploring the underlying processes of the interventions or validating the intervention effects with objective measurements |
| 30 | to investigate the role of feedback from the gaming console on outcomes |
| 31 | longer-term effects |
| 32 | test the effectiveness of the apps in institutional contexts and in participants with tablet experience; develop and test the efficacy of apps that use social support and tailoring to increase fruit and vegetable intake |
| 33 | different exercise types should be compared to exergaming |
| 34 | video components of interventions to prompt PA in older adults, longer-term effects, add-on to clinical therapy |
| 35 | development and implementation of digital interventions in rural areas |
| 36 | objective measures of health; characteristics of messages (frequency, style, time sent). |
| 37 | long-term effectiveness, the confouding of effects due to other activities performed during the study duration, comparison among different forms of exergaming (with high vs. low playfulness) |
| 38 | data visualization in wearables to meet the preferences of older adults, effectiveness of different wearables, engagement with devices, options for sharing device data with others (eg, clinicians, family) |
| 39 | long-term effectivness |
| 40 | application of eHealth interventions in low income countries, objective measures of behavior, factors that could improve long-term effectiveness |
| 41 | effectiveness of cognitive monitoring in participants with cognitive decline |
| 42 | intervention delivery via other devices, eg, smartphones, long-term effects, objective outcome measurement, self-regulation factors that could reduce attrition |
| 43 | effectivnesss of playing with peers of different age groups (eg, young >75 vs. old 75+ age groups) or familiarity with peers |
| 44 | implementation of digital interventions as a service with human support and not as a stand-alone-program |
| 45 | how components of intervention (social app use, coaching) contribute to effectiveness of interventions |
| 46 | replicability in other health conditions, long-term effects, cost-effectivness, objective measures of behavior |
| 47 | other measures of balance, controlling for confouding by other activities between training sessions |
| 48 | intervention features needed to improve website usage and optimise PA outcomes in older adults, long-term maintenance |
| 49 | efficacy of wearables should be tested; desire for integration of this intervention with the medical system |
| 50 | motivation and engagment factors to participate in the virtual community long-term |
| 51 | testing the use of wearable devices to improve health in frail people |
| 52 | effectiveness of different components of the intervention, in the long-term and compared to a control group |
| 53 | test the effectiveness of text messages in less active older adults |
| 54 | using a tablet with larger screen, clinical application (blended care) |
| 55 | long-term effects; exercise programs providing a greater variety of games with increasing levels of difficulty; providing on-screen step-by-step instructions |
| 56 | effectiveness and implementation in clinical settings and low-income settings |
| 57 | focus on mental health needs to encourage socialization and PA |
| 58 | exploring what severity of depression or anxiety causes disengagement with technology and in what populations this is most common, the use of prompts from digital devices to improve engagement, technology guidance |
| 59 | strategies to improve the adherence to interventions deployed in the daily life of older adults to support prevention of functional decline |
| 60 | transfer to daily life: focus on interventions that motivate elderly people in their communities to walk together, to strengthen their social networks and, consequently, to positively impact their health beyond the study period |
| 61 | Learning effects to operate the app, usefulness, and long-term satisfaction with the app |
| 62 | influence of different modes of delivery and user preferences on participation and effects |
| 63 | factors required to improve the interest and acceptance of technology among older people |
| 64 | optimal dose of computerized cognitive training or other intervention activities needed for optimal preventive effects |
| 65 | long-term effects, other factors that may confound the PA outcomes during the study (eg, buying a new bike) |
| 66 | understand the mechanisms of how wearables can improve sleep measures |
| 67 | future studies should include more ecological assessment instruments and follow-up evaluations; effects of sample characteristics, the programme employed, programme duration and format on effectivness of exergaming |
| 68 | long-term effectivness and maintenance of effects in the absence of intervention |
| 69 | better understand the predictors of technology use in the aging world, clinical application; a culture shift in the provision of care to digital platforms, especially in the context of COVID |
| 70 | to evaluate adherence to new digital fall prevention interventions and consistency in the way adherence is assessed |
| 71 | Assess acceptance after use, social influences on acceptability, longitudinal study focusing on acceptance |
| 72 | clinical effectiveness, long-term effects, tailoring toward user´s preferences |
| 73 | Tailoring of games (concerning pace and level of knowledge content), identification of home environment and network conditions most suitable for connected health interventions for older adults living in the community |
| 74 | mindfulness effect on intentionality to engage in PA, long-term effects |
| 75 | development and testing of virtual delivery systems that can counteract forgetfulness on how to perform exercise |
| 76 | clinical usability; provide a personalized approach to treatment using data from digital devices, appropriate intensity of intervention to enhance longer-term intervention compliance |
| 77 | further research could help understand mechanisms by which technology can improve distal outcomes of weight loss and physical function, a longer study is needed both in terms of short-term weight loss but also in terms of weight maintenance strategies, comparing interventions with and without technology augmentation |
| 78 | evaluate strategies for replacing sedentary time with standing and PA |
| 79 | self-efficacy should be measured in future interventions |
| 80 | economic evaluation |
| 81 | types of engagement (e.g., patterns of engagement over time), ways of operationalizing engagement that better capture unique user behavior patterns or trends, testing fully digital interventions in older populations using non-observational designs |
| 82 | different exercise doses |
| 83 | attitudes to digital technologie in samples with less digital competence |
| 84 | clinical effectiveness |
| 85 | Future research should continue investigating the utility and efficacy of telehealth interventions in older populations, especially for those living with muscle dysfunction and morbidities that can undermine independence such as obesity. Larger sample sizes, stronger experimental designs with follow up and control groups, and additional objective measures should also be considered for this translational research when possible. |
| 86 | leisure PA may confound the results collected from the wearable device |
| 87 | “The potential negative effects that might be due to feelings of inadequacy or failure dues to upward social comparisons (Wang et al., 2017) should be considered in future research.” “Further research is needed to understand under what circumstances social comparison in social networks is related to positive or negative outcomes, especially among older adults." |
| 88 | consider participant preferences and perspectives regarding privacy, data confidentiality, desire to provide permission, and sharing study  results when designing mHealth interventions |
| 89 | design of interventions to promote PA among cancer survivors, Audiobooks offered through a community resource also allows for sustainability  of this component of the intervention |
| 90 | “user-centric” approach, leveraging both qualitative data (e.g., focus groups and survey work) and other data to identify key aspects of mobile programs that present challenges, in addition to attractive features that would be of most interest/utility to older users; long term health outcomes |
| 91 | long-term effects on learning and behavior change |
| 92 | to find out how to best itegrate addditional information (eg, environmental information) in health interventions promoting physical activity |
| 93 | factors explaining and stimulating the maintenance of the Web-based version, validation of self-reported data with objective measurements |
| 94 | confounding of the results of interventions based on baseline PA level; objective measures of compliance |
| 95 | mechnisms of the relationship between PA and quality of life in inactive older adults (inactivity associated with problems with daily functioning); clinical application of the intervention |
| 96 | assess the interaction between computer skills and intervention effectivness, if the learned computer skills are retained by older adults in the long-term |
| 97 | explore the potential for using technology for health promotion among older adults from low-income groups, underrepresented minorities, and among those with no prior exposure to online or other digital technologies |
| 98 | diverse older adult populations; effectiveness of the intervention |
| 99 | clinical application of the intervention |
| 100 | designs to reduce health inequalities when implementing health interventions |
| 101 | self-monitoring as a further mode of tailoring interventions, longer follow-up, effects on different types of PA in disadvantaged populations with health impairments |
| 102 | identification of tailored requirements of subgroups |
| 103 | evaluation of app effectiveness |
| 104 | explore the views of participants who discontinued the digital intervention |
| 105 | trust-building factors in technology use; underlying facilitators  supporting the engagement of older adult users; social aspects of AI-based technology facilitating social interactions needed in aging; focus on design and testing of digital technologies for health promotion and disease prevention rather than for clinical treatment |

## Textbox S3. Overlap among primary studies.

| **Overlap in primary studies included in 8 systematic reviews**   - 88 primary studies included in 8 systematic reviews. - 61/88 unique primary studies included in 8 systematic reviews.   - 38/61 (62.3%) studies included ×1   - 19/61 (31.1%) studies included ×2   - 4/61 (6.6%) studies included ×3   **Overlap in primary studies included in 8 systematic reviews and our scoping review**   - 61/88 unique primary studies included in 8 systematic reviews.   - 44/61 (72.13%) studies included only in any of the 8 systematic reviews but not in our electronic search   - 17/61 (27.87%) studies included in any of the 8 systematic reviews and in our scoping review |
| --- |

## References

1. Alley SJ, Kolt GS, Duncan MJ, Caperchione CM, Savage TN, Maeder AJ, et al. The effectiveness of a web 2.0 physical activity intervention in older adults - a randomised controlled trial. International Journal of Behavioral Nutrition & Physical Activity. 2018 01 12;15(1):4. doi: <https://dx.doi.org/10.1186/s12966-017-0641-5>.

2. Ammann R, Vandelanotte C, de Vries H, Mummery WK. Can a Website-Delivered Computer-Tailored Physical Activity Intervention Be Acceptable, Usable, and Effective for Older People? Health Education & Behavior. 2012 2013/04/01;40(2):160-70. doi: 10.1177/1090198112461791.

3. Andrews JA, Brown LJ, Hawley MS, Astell AJ. Older adults’ perspectives on using digital technology to maintain good mental health: interactive group study. Journal of Medical Internet Research. 2019;21(2):e11694.

4. Antoine Parker C, Ellis R. Effect of Electronic Messaging on Physical Activity Participation among Older Adults. J Aging Res. 2016;2016:6171028. doi: 10.1155/2016/6171028.

5. Auster-Gussman LA, Lockwood KG, Graham SA, Pitter V, Branch OH. Engagement in Digital Health App-Based Prevention Programs Is Associated With Weight Loss Among Adults Age 65+. Frontiers in Digital Health. 2022 2022-May-19;4. doi: 10.3389/fdgth.2022.886783.

6. Batsis JA, Dokko R, Naslund JA, Zagaria AB, Kotz D, Bartels SJ, et al. Opportunities to Improve a Mobile Obesity Wellness Intervention for Rural Older Adults with Obesity. Journal of Community Health. 2020;45(1):194-200. doi: 10.1007/s10900-019-00720-y.

7. Batsis JA, Naslund JA, Gill LE, Masutani RK, Agarwal N, Bartels SJ. Use of a Wearable Activity Device in Rural Older Obese Adults: A Pilot Study. Gerontol Geriatr Med. 2016 Jan-Dec;2:2333721416678076. doi: 10.1177/2333721416678076.

8. Batsis JA, Naslund JA, Zagaria AB, Kotz D, Dokko R, Bartels SJ, et al. Technology for Behavioral Change in Rural Older Adults with Obesity. Journal of Nutrition in Gerontology & Geriatrics. 2019 Apr-Jun;38(2):130-48. doi: <https://dx.doi.org/10.1080/21551197.2019.1600097>.

9. Batsis JA, Petersen CL, Clark MM, Cook SB, Kotz D, Gooding TL, et al. Feasibility and acceptability of a technology-based, rural weight management intervention in older adults with obesity. BMC Geriatrics. 2021;21(1):44-. doi: 10.1186/s12877-020-01978-x.

10. Batsis JA, Petersen CL, Clark MM, Cook SB, Lopez-Jimenez F, Al-Nimr RI, et al. A Weight Loss Intervention Augmented by a Wearable Device in Rural Older Adults With Obesity: A Feasibility Study. Journals of Gerontology Series A: Biological Sciences & Medical Sciences. 2021;76(1):95-100. doi: 10.1093/gerona/glaa115.

11. Batsis JA, Zagaria A, Kotz DF, Bartels SJ, Boateng GG, Proctor PO, et al. Usability evaluation for the Amulet Wearable Device in rural older adults with obesity. Gerontechnology. 2018;17(3):151-9. doi: 10.4017/gt.2018.17.3.003.00.

12. Bickmore TW, Caruso L, Clough-Gorr K, Heeren T. ‘It's just like you talk to a friend’ relational agents for older adults. Interacting with Computers. 2005 2005/12/01/;17(6):711-35. doi: <https://doi.org/10.1016/j.intcom.2005.09.002>.

13. Blair CK, Harding E, Wiggins C, Kang H, Schwartz M, Tarnower A, et al. A Home-Based Mobile Health Intervention to Replace Sedentary Time With Light Physical Activity in Older Cancer Survivors: Randomized Controlled Pilot Trial. JMIR Cancer. 2021 Apr 13;7(2):e18819. doi: <https://dx.doi.org/10.2196/18819>.

14. Botner E. Impact of a Virtual Learning Program on Social Isolation for Older Adults. Therapeutic Recreation Journal. 2018;52(2):126-39. doi: 10.18666/TRJ-2018-V52-I2-8664.

15. Broekhuizen K, de Gelder J, Wijsman CA, Wijsman LW, Westendorp RG, Verhagen E, et al. An Internet-Based Physical Activity Intervention to Improve Quality of Life of Inactive Older Adults: A Randomized Controlled Trial. Journal of Medical Internet Research. 2016 Apr 27;18(4):e74. doi: <https://dx.doi.org/10.2196/jmir.4335>.

16. Cabrita M, Tabak M, Vollenbroek-Hutten MRM. Older Adults? Attitudes Toward Ambulatory Technology to Support Monitoring and Coaching of Healthy Behaviors: Qualitative Study. JMIR Aging. 2019 03/12;2(1):e10476.

17. Cadmus-Bertram LA, Marcus BH, Patterson RE, Parker BA, Morey BL. Randomized Trial of a Fitbit-Based Physical Activity Intervention for Women. Am J Prev Med. 2015 Sep;49(3):414-8. doi: 10.1016/j.amepre.2015.01.020.

18. Carrasco M, Ortiz-Maques N, Martinez-Rodriguez S. Playing with Nintendo Wii Sports: Impact on physical activity, perceived health and cognitive functioning of a group of community-dwelling older adults. Activities, Adaptation & Aging. 2020 Apr-Jun;44(2):119-31. doi: <https://dx.doi.org/10.1080/01924788.2019.1595261>.

19. Coley N, Andre L, Hoevenaar-Blom MP, Ngandu T, Beishuizen C, Barbera M, et al. Factors Predicting Engagement of Older Adults With a Coach-Supported eHealth Intervention Promoting Lifestyle Change and Associations Between Engagement and Changes in Cardiovascular and Dementia Risk: Secondary Analysis of an 18-Month Multinational Randomized Controlled Trial. Journal of medical Internet research. 2022;24(5):e32006.

20. Compernolle S, Cardon G, van der Ploeg HP, Van Nassau F, De Bourdeaudhuij I, Jelsma JJ, et al. Engagement, Acceptability, Usability, and Preliminary Efficacy of a Self-Monitoring Mobile Health Intervention to Reduce Sedentary Behavior in Belgian Older Adults: Mixed Methods Study. JMIR MHealth and UHealth. 2020 10 29;8(10):e18653. doi: <https://dx.doi.org/10.2196/18653>.

21. Cook FR, Hersch KR, Schlossberg D, Leaf LS. A Web-Based Health Promotion Program for Older Workers: Randomized Controlled Trial. J Med Internet Res. 2015 03/25;17(3):e82.

22. Cooper D, Kavanagh R, Bolton J, Myers C, O'Connor S. 'Prime time of life', a 12-week home-based online multimodal exercise training and health education programme for middle-aged and older adults in laois. Physical Activity and Health. 2021;5(1):178-94. doi: 10.5334/PAAH.122.

23. Corbett A, Owen A, Hampshire A, Grahn J, Stenton R, Dajani S, et al. The Effect of an Online Cognitive Training Package in Healthy Older Adults: An Online Randomized Controlled Trial. Journal of the American Medical Directors Association. 2015 Nov 01;16(11):990-7. doi: <https://dx.doi.org/10.1016/j.jamda.2015.06.014>.

24. David P, Buckworth J, Pennell ML, Katz ML, Degraffinreid CR, Paskett ED. A walking intervention for postmenopausal women using mobile phones and Interactive Voice Response. Journal of Telemedicine & Telecare. 2012;18(1):20-5. doi: 10.1258/jtt.2011.110311.

25. Dekker-van Weering M, Jansen-Kosterink S, Frazer S, Vollenbroek-Hutten M. User Experience, Actual Use, and Effectiveness of an Information Communication Technology-Supported Home Exercise Program for Pre-Frail Older Adults. Front Med (Lausanne). 2017;4:208. doi: 10.3389/fmed.2017.00208.

26. Delbaere K, Valenzuela T, Lord SR, Clemson L, Zijlstra GAR, Close JCT, et al. E-health StandingTall balance exercise for fall prevention in older people: Results of a two year randomised controlled trial. The BMJ. 2021;373. doi: 10.1136/bmj.n740.

27. Frei A, Dalla Lana K, Radtke T, Stone E, Knöpfli N, Puhan MA. A novel approach to increase physical activity in older adults in the community using citizen science: a mixed-methods study. International Journal of Public Health (Springer Nature). 2019;64(5):669-78. doi: 10.1007/s00038-019-01230-3.

28. Graham SA, Stein N, Shemaj F, Branch OH, Paruthi J, Kanick SC. Older Adults Engage With Personalized Digital Coaching Programs at Rates That Exceed Those of Younger Adults. Frontiers in Digital Health. 2021;3:642818. doi: <https://dx.doi.org/10.3389/fdgth.2021.642818>.

29. Gschwind YJ, Eichberg S, Ejupi A, de Rosario H, Kroll M, Marston HR, et al. ICT-based system to predict and prevent falls (iStoppFalls): results from an international multicenter randomized controlled trial. Eur Rev Aging Phys Act. 2015;12:10. doi: 10.1186/s11556-015-0155-6.

30. Haeger M, Bock O, Zijlstra W. [Smartphone-based health promotion in old age : An explorative multi-component approach to improving health in old age]. Zeitschrift fur Gerontologie und Geriatrie. 2021 Mar;54(2):146-51. doi: <https://dx.doi.org/10.1007/s00391-020-01700-x>.

31. Hageman PA, Walker SN, Pullen CH. Tailored versus standard Internet-delivered interventions to promote physical activity in older women. Journal of Geriatric Physical Therapy. 2005;28(1):28-33.

32. Ienca M, Schneble C, Kressig RW, Wangmo T. Digital health interventions for healthy ageing: a qualitative user evaluation and ethical assessment. BMC Geriatrics. 2021;21(1):1-10. doi: 10.1186/s12877-021-02338-z.

33. Irvine AB, Gelatt VA, Seeley JR, Macfarlane P, Gau JM. Web-based intervention to promote physical activity by sedentary older adults: randomized controlled trial. Journal of Medical Internet Research. 2013;15(2):e19-e. doi: 10.2196/jmir.2158.

34. Jaana M, Paré G. Comparison of mobile health technology use for Self-Tracking between older adults and the general adult population in Canada: cross-sectional survey. JMIR mHealth and uHealth. 2020;8(11):e24718.

35. Jacobson CL, Foster LC, Arul H, Rees A, Stafford RS. A Digital Health Fall Prevention Program for Older Adults: Feasibility Study. JMIR Formative Research. 2021;5(12). doi: 10.2196/30558.

36. Jang IY, Kim HR, Lee E, Jung HW, Park H, Cheon SH, et al. Impact of a wearable device-based walking programs in rural older adults on physical activity and health outcomes: Cohort study. JMIR mHealth and uHealth. 2018;6(11). doi: 10.2196/11335.

37. Jenaro C, Flores N, Cruz M, Moro L, Pérez C. Efficacy of text messaging for health care in the elderly (Eficacia de los mensajes de texto para el cuidado de la salud en población mayorn mayor). Gerokomos. 2016;27(2):42-7.

38. Johnson N, Bradley A, Klawitter L, Johnson J, Johnson L, Tomkinson GR, et al. The impact of a telehealth intervention on activity profiles in older adults during the COVID-19 pandemic: A pilot study. Geriatrics. 2021;6(3). doi: 10.3390/geriatrics6030068.

39. Kahlbaugh PE, Sperandio AJ, Carlson AL, Hauselt J. Effects of Playing Wii on Well-Being in the Elderly: Physical Activity, Loneliness, and Mood. Activities, Adaptation & Aging. 2011 2011/10/01;35(4):331-44. doi: 10.1080/01924788.2011.625218.

40. Kim BH, Glanz K. Text messaging to motivate walking in older african americans: A randomized controlled trial. American Journal of Preventive Medicine. 2013;44(1):71-5. doi: 10.1016/j.amepre.2012.09.050.

41. King AC, Bickmore TW, Campero MI, Pruitt LA, Yin JL. Employing virtual advisors in preventive care for underserved communities: results from the COMPASS study. Journal of Health Communication. 2013;18(12):1449-64. doi: 10.1080/10810730.2013.798374.

42. Kirk A, MacMillan F, Rice M, Carmichael A. An exploratory study examining the appropriateness and potential benefit of the Nintendo Wii as a physical activity tool in adults aged >= 55 years. Interacting with Computers. 2013 Jan;25(1):102-14.

43. Kumar S, Tran JLA, Moseson H, Tai C, Glenn JM, Madero EN, et al. The impact of the virtual cognitive health program on the cognition and mental health of older adults: Pre-Post 12-Month pilot study. JMIR Aging. 2018;1(2). doi: 10.2196/12031.

44. Kurti AN, Dallery J. Internet-based contingency management increases walking in sedentary adults. Journal of Applied Behavior Analysis. 2013;46(3):568-81. doi: <https://dx.doi.org/10.1002/jaba.58>.

45. Lee HY, Kim J, Kim KS. The Effects of Nursing Interventions Utilizing Serious Games That Promote Health Activities on the Health Behaviors of Seniors. Games for Health Journal. 2015 Jun;4(3):175-82. doi: <https://dx.doi.org/10.1089/g4h.2014.0124>.

46. Lee WJ, Peng LN, Lin MH, Loh CH, Chen LK. Active wearable device utilization improved physical performance and IGF-1 among community-dwelling middle-aged and older adults: a 12-month prospective cohort study. Aging. 2021 08 03;13(15):19710-21. doi: <https://dx.doi.org/10.18632/aging.203383>.

47. Li J, Theng Y-L, Foo S. Exergames for older adults with subthreshold depression: Does higher playfulness lead to better improvement in depression? Games for Health. 2016 Jun;5(3):175-82. doi: <https://dx.doi.org/10.1089/g4h.2015.0100>.

48. Liu Y, Lachman ME. A Group-Based Walking Study to Enhance Physical Activity Among Older Adults: The Role of Social Engagement. Research on Aging. 2021;43(9/10):368-77. doi: 10.1177/0164027520963613.

49. Lyons EJ, Swartz MC, Lewis ZH, Martinez E, Jennings K. Feasibility and Acceptability of a Wearable Technology Physical Activity Intervention With Telephone Counseling for Mid-Aged and Older Adults: A Randomized Controlled Pilot Trial. JMIR Mhealth Uhealth. 2017 Mar 6;5(3):e28. doi: 10.2196/mhealth.6967.

50. Mansson L, Lundin-Olsson L, Skelton DA, Janols R, Lindgren H, Rosendahl E, et al. Older adults' preferences for, adherence to and experiences of two self-management falls prevention home exercise programmes: a comparison between a digital programme and a paper booklet. BMC Geriatr. 2020 Jun 15;20(1):209. doi: 10.1186/s12877-020-01592-x.

51. Mascret N, Delbes L, Voron A, Temprado J-J, Montagne G. Acceptance of a Virtual Reality Headset Designed for Fall Prevention in Older Adults: Questionnaire Study. J Med Internet Res. 2020 12/14;22(12):e20691.

52. McMahon KS, Lewis B, Oakes M, Guan W, Wyman FJ, Rothman JA. Older Adults Experiences Using a Commercially Available Monitor to Self-Track Their Physical Activity. JMIR mHealth uHealth. 2016 04/13;4(2):e35.

53. McMahon S, Vankipuram M, Hekler EB, Fleury J. Design and evaluation of theory-informed technology to augment a wellness motivation intervention. Translational Behavioral Medicine. 2014 Mar;4(1):95-107. doi: <https://dx.doi.org/10.1007/s13142-013-0221-4>.

54. McMahon SK, Wyman JF, Belyea MJ, Shearer N, Hekler EB, Fleury J. Combining Motivational and Physical Intervention Components to Promote Fall-Reducing Physical Activity Among Community-Dwelling Older Adults: A Feasibility Study. American Journal of Health Promotion. 2016;30(8):638-44. doi: 10.4278/ajhp.130522-ARB-265.

55. Mehra S, Visser B, Cila N, van den Helder J, Engelbert RH, Weijs PJ, et al. Supporting Older Adults in Exercising With a Tablet: A Usability Study. JMIR Hum Factors. 2019 Feb 1;6(1):e11598. doi: 10.2196/11598.

56. Mouton A, Cloes M. Efficacy of a web-based, center-based or combined physical activity intervention among older adults. Health Education Research. 2015;30(3):422-35. doi: her/cyv012.

57. Muellmann S, Buck C, Voelcker-Rehage C, Bragina I, Lippke S, Meyer J, et al. Effects of two web-based interventions promoting physical activity among older adults compared to a delayed intervention control group in Northwestern Germany: Results of the PROMOTE community-based intervention trial. Preventive Medicine Reports. 2019 2019/09/01/;15:100958. doi: <https://doi.org/10.1016/j.pmedr.2019.100958>.

58. Müller AM, Khoo S, Morris T. Text Messaging for Exercise Promotion in Older Adults From an Upper-Middle-Income Country: Randomized Controlled Trial. Journal of Medical Internet Research. 2016;18(1):1-. doi: 10.2196/jmir.5235.

59. Nahm E-S, Barker B, Resnick B, Covington B, Magaziner J, Brennan PF. Effects of a social cognitive theory-based hip fracture prevention Web site for older adults. CIN: Computers, Informatics, Nursing. 2010;28(6):371-9. doi: 10.1097/NCN.0b013e3181f69d73.

60. Nahm ES, Resnick B, Brown C, Zhu S, Magaziner J, Bellantoni M, et al. The Effects of an Online Theory-Based Bone Health Program for Older Adults. Journal of Applied Gerontology. 2017 09;36(9):1117-44. doi: <https://dx.doi.org/10.1177/0733464815617284>.

61. Nahm ES, Resnick B, DeGrezia M, Brotemarkle R. Use of discussion boards in a theory-based health web site for older adults. Nurs Res. 2009 Nov-Dec;58(6):419-26. doi: 10.1097/NNR.0b013e3181bee6c4.

62. Nebeker C, Zlatar ZZ. Learning From Older Adults to Promote Independent Physical Activity Using Mobile Health (mHealth). Frontiers in Public Health. 2021;9:703910. doi: <https://dx.doi.org/10.3389/fpubh.2021.703910>.

63. O'Brien T, Jenkins C, Amella E, Mueller M, Moore M, Hathaway D. An Internet-Assisted Weight Loss Intervention for Older Overweight and Obese Rural Women: A feasibility study. CIN: Computers, Informatics, Nursing. 2016;34(11):513-9. doi: 10.1097/cin.0000000000000275.

64. O'Brien TR, Jenkins C, Amella E, Mueller M, Moore M, Troutman-Jordan M, et al. Perceptions of older rural women using computerized programs for weight. Online Journal of Rural Nursing & Health Care. 2014;14(2). doi: <http://dx.doi.org/10.14574/ojrnhc.v14i2.324>.

65. Padala KP, Padala PR, Lensing SY, Dennis RA, Bopp MM, Parkes CM, et al. Efficacy of Wii-Fit on Static and Dynamic Balance in Community Dwelling Older Veterans: A Randomized Controlled Pilot Trial. Journal of Aging Research. 2017;2017. doi: 10.1155/2017/4653635.

66. Papi E, Chiou S-Y, McGregor AH. Feasibility and acceptability study on the use of a smartphone application to facilitate balance training in the ageing population. BMJ open. 2020;10(12):e039054-e. doi: 10.1136/bmjopen-2020-039054.

67. Peels DA, Bolman C, Golsteijn RH, de Vries H, Mudde AN, van Stralen MM, et al. Long-term efficacy of a printed or a Web-based tailored physical activity intervention among older adults. International Journal of Behavioral Nutrition & Physical Activity. 2013 Sep 02;10:104. doi: <https://dx.doi.org/10.1186/1479-5868-10-104>.

68. Peels DA, de Vries H, Bolman C, Golsteijn RH, van Stralen MM, Mudde AN, et al. Differences in the use and appreciation of a web-based or printed computer-tailored physical activity intervention for people aged over 50 years. Health Education Research. 2013 Aug;28(4):715-31. doi: <https://dx.doi.org/10.1093/her/cyt065>.

69. Peels DA, van Stralen MM, Bolman C, Golsteijn RH, de Vries H, Mudde AN, et al. The differentiated effectiveness of a printed versus a Web-based tailored physical activity intervention among adults aged over 50. Health Education Research. 2014 Oct;29(5):870-82. doi: <https://dx.doi.org/10.1093/her/cyu039>.

70. Pettersson B, Janols R, Wiklund M, Lundin-Olsson L, Sandlund M. Older Adults Experiences of Behavior Change Support in a Digital Fall Prevention Exercise Program: Qualitative Study Framed by the Self-determination Theory. J Med Internet Res. 2021 7/30;23(7):e26235.

71. Pinto BM, Kindred M, Franco R, Simmons V, Hardin J. A 'novel' multi-component approach to promote physical activity among older cancer survivors: a pilot randomized controlled trial. Acta Oncologica. 2021;60(8):968-75. doi: 10.1080/0284186X.2021.1896032.

72. Proyer RT, Gander F, Wellenzohn S, Ruch W. Positive psychology interventions in people aged 50–79 years: long-term effects of placebo-controlled online interventions on well-being and depression. Aging & Mental Health. 2014;18(8):997-1005. doi: 10.1080/13607863.2014.899978.

73. Pullen CH, Hageman PA, Boeckner L, Walker SN, Oberdorfer MK. Feasibility of Internet-delivered weight loss interventions among rural women ages 50-69. Journal of Geriatric Physical Therapy. 2008;31(3):105-12.

74. Radhakrishnan K, Julien C, O'Hair M, Baranowski T, Lee G, Allen C, et al. Usability Testing of a Sensor-Controlled Digital Game to Engage Older Adults with Heart Failure in Physical Activity and Weight Monitoring. Applied Clinical Informatics. 2020 10;11(5):873-81. doi: <https://dx.doi.org/10.1055/s-0040-1721399>.

75. Ratz T, Lippke S, Muellmann S, Peters M, Pischke CR, Meyer J, et al. Effects of Two Web-Based Interventions and Mediating Mechanisms on Stage of Change Regarding Physical Activity in Older Adults. Applied Psychology Health and Well-being. 2020 03;12(1):77-100. doi: <https://dx.doi.org/10.1111/aphw.12174>.

76. Richard E, Moll van Charante EP, Hoevenaar-Blom MP, Coley N, Barbera M, van der Groep A, et al. Healthy ageing through internet counselling in the elderly (HATICE): a multinational, randomised controlled trial. The Lancet Digital Health. 2019;1(8):e424-e34. doi: 10.1016/S2589-7500(19)30153-0.

77. Robin N, Toussaint L, Coudevylle GR, Ruart S, Hue O, Sinnapah S. Text Messages Promoting Mental Imagery Increase Self-Reported Physical Activity in Older Adults: A Randomized Controlled Study. Journal of Aging & Physical Activity. 2018;26(3):462-70. doi: 10.1123/japa.2017-0069.

78. Robin N, Toussaint L, Sinnapah S, Hue O, Coudevylle GR. Beneficial Influence of Mindfulness Training Promoted by Text Messages on Self-Reported Aerobic Physical Activity in Older Adults: A Randomized Controlled Study. Journal of Aging & Physical Activity. 2020;28(3):406-14. doi: 10.1123/japa.2019-0002.

79. Sato K, Kuroki K, Saiki S, Nagatomi R. The effects of exercise intervention using Kinect TM on healthy elderly individuals: A quasi-experimental study. Open Journal of Therapy and Rehabilitation. 2014;2(1):38-44. doi: <http://dx.doi.org/10.4236/ojtr.2014.21008>.

80. Sato K, Kuroki K, Saiki S, Nagatomi R. Improving walking, muscle strength, and balance in the elderly with an exergame using Kinect: A randomized controlled trial. Games for Health. 2015 Jun;4(3):161-7. doi: <https://dx.doi.org/10.1089/g4h.2014.0057>.

81. Shubert TE, Chokshi A, Mendes VM, Grier S, Buchanan H, Basnett J, et al. Stand Tall--A Virtual Translation of the Otago Exercise Program. Journal of Geriatric Physical Therapy. 2020;43(3):120-7. doi: 10.1519/JPT.0000000000000203.

82. Sill J, Steenbock B, Helmer S, Zeeb H, Pischke CR. Apps zur Förderung von körperlicher Aktivität – Nutzung und Einstellungen bei Erwachsenen im Alter von 50 Jahren und älter: Ergebnisse eines Online-Surveys (Apps for the promotion of physical activity—use and attitudes among adults aged 50 years and older: Results of an online survey). Prävention und Gesundheitsförderung. 2019 2019/05/01;14(2):109-18. doi: 10.1007/s11553-018-0674-x.

83. Silveira P, van de Langenberg R, van Het Reve E, Daniel F, Casati F, de Bruin ED. Tablet-based strength-balance training to motivate and improve adherence to exercise in independently living older people: a phase II preclinical exploratory trial. J Med Internet Res. 2013 Aug 12;15(8):e159. doi: 10.2196/jmir.2579.

84. Similä H, Immonen M, Toska-Tervola J, Enwald H, Keränen N, Kangas M, et al. Feasibility of mobile mental wellness training for older adults. Geriatric Nursing. 2018;39(5):499-505. doi: 10.1016/j.gerinurse.2018.02.001.

85. Strand KA, Francis SL, Margrett JA, Franke WD, Peterson MJ. Community-based exergaming program increases physical activity and perceived wellness in older adults. Journal of Aging & Physical Activity. 2014 Jul;22(3):364-71. doi: <https://dx.doi.org/10.1123/japa.2012-0302>.

86. Tam E, Boas PKV, Ruaro F, Flesch J, Wu J, Thomas A, et al. Feasibility and Adoption of a Focused Digital Wellness Program in Older Adults. Geriatrics. 2021 May 19;6(2):19. doi: <https://dx.doi.org/10.3390/geriatrics6020054>.

87. Tiedemann A, Hassett L, Sherrington C. A novel approach to the issue of physical inactivity in older age. Preventive Medicine Reports. 2015;2:595-7. doi: 10.1016/j.pmedr.2015.07.008.

88. Tse MM, Choi KC, Leung RS. E-health for older people: the use of technology in health promotion. Cyberpsychology & Behavior. 2008 Aug;11(4):475-9. doi: <https://dx.doi.org/10.1089/cpb.2007.0151>.

89. Turunen M, Hokkanen L, Bäckman L, Stigsdotter-Neely A, Hänninen T, Paajanen T, et al. Computer-based cognitive training for older adults: Determinants of adherence. PLoS One. 2019;14(7):e0219541. doi: 10.1371/journal.pone.0219541.

90. Valdes EG, Sadeq NA, Bush AL, Morgan D, Andel R. Regular cognitive self-monitoring in community-dwelling older adults using an internet-based tool. Journal of Clinical and Experimental Neuropsychology. 2016 Oct;38(9):1026-37. doi: <https://dx.doi.org/10.1080/13803395.2016.1186155>.

91. Valenzuela T, Razee H, Schoene D, Lord RS, Delbaere K. An Interactive Home-Based Cognitive-Motor Step Training Program to Reduce Fall Risk in Older Adults: Qualitative Descriptive Study of Older Adults Experiences and Requirements. JMIR Aging. 2018 11/30;1(2):e11975.

92. van der Mark M, Jonasson J, Svensson M, Linné Y, Rossner S, Lagerros YT. Older members perform better in an internet-based behavioral weight loss program compared to younger members. Obes Facts. 2009;2(2):74-9. doi: 10.1159/000209383.

93. Van Dyck D, Herman K, Poppe L, Crombez G, Bourdeaudhuij ID, Gheysen F, et al. Results of MyPlan 2.0 on Physical Activity in Older Belgian Adults: Randomized Controlled Trial. Journal of Medical Internet Research. 2019;21(10):N.PAG-N.PAG. doi: 10.2196/13219.

94. Van Dyck D, Plaete J, Cardon G, Crombez G, De Bourdeaudhuij I. Effectiveness of the self-regulation eHealth intervention 'MyPlan1.0.' on physical activity levels of recently retired Belgian adults: a randomized controlled trial. Health Education Research. 2016 10;31(5):653-64. doi: <https://dx.doi.org/10.1093/her/cyw036>.

95. van Het Reve E, Silveira P, Daniel F, Casati F, de Bruin ED. Tablet-based strength-balance training to motivate and improve adherence to exercise in independently living older people: part 2 of a phase II preclinical exploratory trial. J Med Internet Res. 2014 Jun 25;16(6):e159. doi: 10.2196/jmir.3055.

96. van Middelaar T, Beishuizen CRL, Guillemont J, Barbera M, Richard E, Moll van Charante EP. Engaging older people in an internet platform for cardiovascular risk self-management: a qualitative study among Dutch HATICE participants. BMJ Open. 2018 Jan 21;8(1):e019683. doi: 10.1136/bmjopen-2017-019683.

97. VanRavenstein K, Davis HB. When More Than Exercise Is Needed to Increase Chances of Aging in Place: Qualitative Analysis of a Telehealth Physical Activity Program to Improve Mobility in Low-Income Older Adults. JMIR Aging. 2018 12/21;1(2):e11955.

98. Watkins I, Xie B. Older adults' perceptions of using iPads for improving fruit and vegetable intake: an exploratory study. Care Management Journals. 2015;16(1):2-13.

99. Wichmann F, Pischke CR, Jürgens D, Darmann-Finck I, Koppelin F, Lippke S, et al. Requirements for (web-based) physical activity interventions targeting adults above the age of 65 years – qualitative results regarding acceptance and needs of participants and non-participants. BMC Public Health. 2020 2020/06/11;20(1):907. doi: 10.1186/s12889-020-08927-8.

100. Wichmann F, Sill J, Hassenstein MJ, Zeeb H, Pischke CR. Apps zur Förderung von körperlicher Aktivität. Einstellungen, Nutzungspräferenzen und Akzeptanz bei Erwachsenen im Alter von 50 Jahren und älter: Ergebnisse von Fokusgruppendiskussionen (Apps for physical activity promotion. Attitudes, acceptance and utilization preferences among adults aged 50 years and above: results of focus group discussions). Prävention und Gesundheitsförderung. 2019 2019/05/01;14(2):93-101. doi: 10.1007/s11553-018-0678-6.

101. Wijsman CA, Westendorp RG, Verhagen EA, Catt M, Slagboom P, de Craen AJ, et al. Effects of a web-based intervention on physical activity and metabolism in older adults: Randomized controlled trial. Journal of Medical Internet Research. 2013 Nov;15(11):20-32. doi: <https://dx.doi.org/10.2196/jmir.2843>.

102. Wu Z, Li J, Theng Y-L. Examining the influencing factors of exercise intention among older adults: A controlled study between exergame and traditional exercise. Cyberpsychology, Behavior, and Social Networking. 2015 Sep;18(9):521-7. doi: <https://dx.doi.org/10.1089/cyber.2015.0065>.

103. Xu X, Li J, Pham TP, Salmon CT, Theng Y-L. Improving psychosocial well-being of older adults through exergaming: The moderation effects of intergenerational communication and age cohorts. Games for Health. 2016 Dec;5(6):389-97. doi: <https://dx.doi.org/10.1089/g4h.2016.0060>.

104. Yardley L, Nyman SR. Internet provision of tailored advice on falls prevention activities for older people: a randomized controlled evaluation. Health Promotion International. 2007 Jun;22(2):122-8.

105. Zaslavsky O, Thompson HJ, McCurry SM, Landis CA, Kitsiou S, Ward TM, et al. Use of a Wearable Technology and Motivational Interviews to Improve Sleep in Older Adults With Osteoarthritis and Sleep Disturbance: A Pilot Study. Research in Gerontological Nursing. 2019;12(4):167-73. doi: 10.3928/19404921-20190319-02.

106. Donath L, Rössler R, Faude O. Effects of Virtual Reality Training (Exergaming) Compared to Alternative Exercise Training and Passive Control on Standing Balance and Functional Mobility in Healthy Community-Dwelling Seniors: A Meta-Analytical Review. Sports Med. 2016 Sep;46(9):1293-309. doi: 10.1007/s40279-016-0485-1.

107. Larsen LH, Schou L, Lund HH, Langberg H. The Physical Effect of Exergames in Healthy Elderly-A Systematic Review. Games Health J. 2013 Aug;2(4):205-12. doi: 10.1089/g4h.2013.0036.

108. Muellmann S, Forberger S, Möllers T, Bröring E, Zeeb H, Pischke CR. Effectiveness of eHealth interventions for the promotion of physical activity in older adults: A systematic review. Preventive Medicine. 2018;108:93-110. doi: 10.1016/j.ypmed.2017.12.026.

109. Nunez de Arenas-Arroyo S, Cavero-Redondo I, Alvarez-Bueno C, Sequi-Dominguez I, Reina-Gutierrez S, Martinez-Vizcaino V. Effect of eHealth to increase physical activity in healthy adults over 55 years: A systematic review and meta-analysis. Scandinavian Journal of Medicine & Science in Sports. 2021 Apr;31(4):776-89. doi: <https://dx.doi.org/10.1111/sms.13903>.

110. Pacheco TBF, De Medeiros CSP, De Oliveira VHB, Vieira ER, De Cavalcanti FAC. Effectiveness of exergames for improving mobility and balance in older adults: A systematic review and meta-analysis. Systematic Reviews. 2020;9(1). doi: 10.1186/s13643-020-01421-7.

111. Song Y, Qu J, Zhang D, Zhang J. Feasibility and Effectiveness of Mobile Phones in Physical Activity Promotion for Adults 50 Years and Older: A Systematic Review. Topics in Geriatric Rehabilitation. 2018;34(3):213-22. doi: 10.1097/TGR.0000000000000197.

112. Stara V, Santini S, Kropf J, D'Amen B. Digital Health Coaching Programs Among Older Employees in Transition to Retirement: Systematic Literature Review. Journal of Medical Internet Research. 2020;22(9):N.PAG-N.PAG. doi: 10.2196/17809.

113. Yerrakalva D, Yerrakalva D, Hajna S, Griffin S. Effects of Mobile Health App Interventions on Sedentary Time, Physical Activity, and Fitness in Older Adults: Systematic Review and Meta-Analysis. Journal of Medical Internet Research. 2019;21(11):N.PAG-N.PAG. doi: 10.2196/14343.

114. Aalbers T, Baars MAE, Rikkert MGMO. Characteristics of effective Internet-mediated interventions to change lifestyle in people aged 50 and older: A systematic review. Ageing Research Reviews. 2011;10(4):487-97. doi: 10.1016/j.arr.2011.05.001.

115. Beishuizen CR, Stephan BC, van Gool WA, Brayne C, Peters RJ, Andrieu S, et al. Web-Based Interventions Targeting Cardiovascular Risk Factors in Middle-Aged and Older People: A Systematic Review and Meta-Analysis. J Med Internet Res. 2016 Mar 11;18(3):e55. doi: 10.2196/jmir.5218.

116. Chu-Ai R, Mahajan N, Visvanathan R, Wilson A. Clinical effectiveness of and attitudes and beliefs of health professionals towards the use of health technology in falls prevention among older adults. International Journal of Evidence-Based Healthcare. 2015;13(4):213-23. doi: 10.1097/XEB.0000000000000029.

117. Dean DAL, Griffith DM, McKissic SA, Cornish EK, Johnson-Lawrence V. Men on the Move-Nashville: Feasibility and Acceptability of a Technology-Enhanced Physical Activity Pilot Intervention for Overweight and Obese Middle and Older Age African American Men. American Journal of Mens Health. 2018 07;12(4):798-811. doi: <https://dx.doi.org/10.1177/1557988316644174>.

118. Elavsky S, Knapova L, Klocek A, Smahel D. Mobile Health Interventions for Physical Activity, Sedentary Behavior, and Sleep in Adults Aged 50 Years and Older: A Systematic Literature Review. Journal of Aging & Physical Activity. 2019;27(4):565-93. doi: 10.1123/japa.2017-0410.

119. Gould CE, Carlson C, Alfaro AJ, Chick CF, Bruce ML, Forman-Hoffman VL. Changes in Quality of Life and Loneliness Among Middle-Aged and Older Adults Participating in Therapist-Guided Digital Mental Health Intervention. Frontiers in Public Health. 2021;9:746904. doi: <https://dx.doi.org/10.3389/fpubh.2021.746904>.

120. Gould CE, Carlson C, Ma F, Forman-Hoffman V, Ranta K, Kuhn E. Effects of Mobile App-Based Intervention for Depression in Middle-Aged and Older Adults: Mixed Methods Feasibility Study. JMIR Formative Research. 2021 Jun 29;5(6):e25808. doi: <https://dx.doi.org/10.2196/25808>.

121. Hughes SL, Seymour RB, Campbell RT, Shaw JW, Fabiyi C, Sokas R. Comparison of Two Health-Promotion Programs for Older Workers. American Journal of Public Health. 2011;101(5):883-90. doi: 10.2105/AJPH.2010.300082.

122. King AC, Hekler EB, Grieco LA, Winter SJ, Sheats JL, Buman MP, et al. Effects of Three Motivationally Targeted Mobile Device Applications on Initial Physical Activity and Sedentary Behavior Change in Midlife and Older Adults: A Randomized Trial. PLoS ONE [Electronic Resource]. 2016;11(6):e0156370. doi: <https://dx.doi.org/10.1371/journal.pone.0156370>.

123. Kwan RYC, Salihu D, Lee PH, Tse M, Cheung DSK, Roopsawang I, et al. The effect of e-health interventions promoting physical activity in older people: a systematic review and meta-analysis. European Reviews of Aging & Physical Activity. 2020;17:7. doi: <https://dx.doi.org/10.1186/s11556-020-00239-5>.

124. Lee SC, Tsai JM, Tsai LY, Liang LJ, Wu CP. Promoting physical activity and reducing frailty of middle-aged and older adults in community: The effects of a health promotion program combining smart phone learning and exercise. International Journal of Gerontology. 2019;13(4):320-4. doi: 10.6890/IJGE.201912_13(4).0011.

125. Miller KJ, Adair BS, Pearce AJ, Said CM, Ozanne E, Morris MM. Effectiveness and feasibility of virtual reality and gaming system use at home by older adults for enabling physical activity to improve health-related domains: a systematic review. Age & Ageing. 2014;43(2):188-95.

126. Reyes A, Qin P, Brown CA. A standardized review of smartphone applications to promote balance for older adults. Disability & Rehabilitation. 2018;40(6):690-6. doi: 10.1080/09638288.2016.1250124.

127. Robert C, Erdt M, Lee J, Cao Y, Naharudin NB, Theng Y-L. Effectiveness of eHealth Nutritional Interventions for Middle-Aged and Older Adults: Systematic Review and Meta-analysis. Journal of Medical Internet Research. 2021;23(5). doi: 10.2196/15649.

128. Sezgin E, Militello LK, Huang Y, Lin S. A scoping review of patient-facing, behavioral health interventions with voice assistant technology targeting self-management and healthy lifestyle behaviors. Translational Behavioral Medicine. 2020 08 07;10(3):606-28. doi: <https://dx.doi.org/10.1093/tbm/ibz141>.

129. Ventura Marra M, Lilly CL, Nelson KR, Woofter DR, Malone J. A Pilot Randomized Controlled Trial of a Telenutrition Weight Loss Intervention in Middle-Aged and Older Men with Multiple Risk Factors for Cardiovascular Disease. Nutrients. 2019 Jan 22;11(2):22. doi: <https://dx.doi.org/10.3390/nu11020229>.

130. Airola E, Rasi P, Outila M. Older people as users and non-users of a video conferencing service for promoting social connectedness and well-being – a case study from Finnish Lapland. Educational Gerontology. 2020;46(5):258-69. doi: 10.1080/03601277.2020.1743008.

131. Bruce ML, Pepin R, Marti CN, Stevens CJ, Choi NG. One Year Impact on Social Connectedness for Homebound Older Adults: Randomized Controlled Trial of Tele-delivered Behavioral Activation Versus Tele-delivered Friendly Visits. American Journal of Geriatric Psychiatry. 2021;29(8):771-6. doi: 10.1016/j.jagp.2021.05.005.

132. Burke L, Lee AH, Pasalich M, Jancey J, Kerr D, Howat P. Effects of a physical activity and nutrition program for seniors on body mass index and waist-to-hip ratio: A randomised controlled trial. Preventive Medicine. 2012;54(6):397-401. doi: 10.1016/j.ypmed.2012.03.015.

133. Chiu CJ, Hu JC, Lo YH, Chang EY. Health Promotion and Disease Prevention Interventions for the Elderly: A Scoping Review from 2015-2019. International Journal of Environmental Research & Public Health [Electronic Resource]. 2020 07 24;17(15):24. doi: <https://dx.doi.org/10.3390/ijerph17155335>.

134. Geraedts H, Zijlstra A, Bulstra SK, Stevens M, Zijlstra W. Effects of remote feedback in home-based physical activity interventions for older adults: A systematic review. Patient Education & Counseling. 2013;91(1):14-24. doi: 10.1016/j.pec.2012.10.018.

135. Hsu HC, Kuo T, Lin JP, Hsu WC, Yu CW, Chen YC, et al. A Cross-Disciplinary Successful Aging Intervention and Evaluation: Comparison of Person-to-Person and Digital-Assisted Approaches. International Journal of Environmental Research & Public Health [Electronic Resource]. 2018 05 04;15(5):04. doi: <https://dx.doi.org/10.3390/ijerph15050913>.

136. Matson TE, Anderson ML, Renz AD, Greenwood-Hickman MA, McClure JB, Rosenberg DE. Changes in Self-Reported Health and Psychosocial Outcomes in Older Adults Enrolled in Sedentary Behavior Intervention Study. American Journal of Health Promotion. 2019;33(7):1053-7. doi: 10.1177/0890117119841405.

137. Saquib J, King AC, Castro CM, Tinker LF, Sims S, Shikany JM, et al. A pilot study combining Go4Life R materials with an interactive voice response system to promote physical activity in older women. Journal of Women & Aging. 2016;28(5):454-62. doi: <https://dx.doi.org/10.1080/08952841.2015.1018065>.

138. Ardo J, Lee J-A, Hildebrand JA, Guijarro D, Ghasemazadeh H, Stromberg A, et al. Codesign of a cardiovascular disease prevention text message bank for older adults. Patient Education and Counseling. 2021 Nov;104(11):2772-84. doi: <https://dx.doi.org/10.1016/j.pec.2021.03.036>.

139. Bakas T, Sampsel D, Israel J, Chamnikar A, Ellard A, Clark JG, et al. Satisfaction and Technology Evaluation of a Telehealth Robotic Program to Optimize Healthy Independent Living for Older Adults. Journal of Nursing Scholarship. 2018;50(6):666-75. doi: 10.1111/jnu.12436.

140. Brivio E, Serino S, Galimberti C, Riva G. Efficacy of a digital education program on life satisfaction and digital self efficacy in older adults: A mixed method study. Annual Review of CyberTherapy and Telemedicine. 2016;14:45-50.

141. Buyl R, Beogo I, Fobelets M, Deletroz C, Van Landuyt P, Dequanter S, et al. E-Health interventions for healthy aging: A systematic review. Systematic Reviews. 2020;9(1). doi: 10.1186/s13643-020-01385-8.

142. Doñate-Martínez A, Ródenas F, Garcés J. Impact of a primary-based telemonitoring programme in HRQOL, satisfaction and usefulness in a sample of older adults with chronic diseases in Valencia (Spain). Archives of Gerontology and Geriatrics. 2016;62:169-75. doi: 10.1016/j.archger.2015.09.008.

143. Esfandiari E, Miller WC, Ashe MC. The Effect of Telehealth Interventions on Function and Quality of Life for Older Adults with Pre-Frailty or Frailty: A Systematic Review and Meta-Analysis. Journal of Applied Gerontology. 2021;40(11):1649-58. doi: 10.1177/0733464820983630.

144. Forsman AK, Nordmyr J, Matosevic T, Park A, Wahlbeck K, McDaid D. Promoting mental wellbeing among older people: Technology-based interventions. Health Promotion International. 2018 Dec;33(6):1042-54. doi: <https://dx.doi.org/10.1093/heapro/dax047>.

145. Harte R, Hall T, Glynn L, Rodriguez-Molinero A, Scharf T, Quinlan LR, et al. Enhancing Home Health Mobile Phone App Usability Through General Smartphone Training: Usability and Learnability Case Study. JMIR Human Factors. 2018 04 26;5(2):e18. doi: <https://dx.doi.org/10.2196/humanfactors.7718>.

146. Huh J, Le T, Reeder B, Thompson HJ, Demiris G. Perspectives on wellness self-monitoring tools for older adults. International Journal of Medical Informatics. 2013;82(11):1092-103. doi: 10.1016/j.ijmedinf.2013.08.009.

147. Kutz D, Shankar K, Connelly K. Making sense of mobile- and web-based wellness information technology: cross-generational study. Journal of Medical Internet Research. 2013;15(6):e83-e. doi: 10.2196/jmir.2124.

148. Lin WY, Chou WC, Tsai TH, Lin CC, Lee MY. Development of a Wearable Instrumented Vest for Posture Monitoring and System Usability Verification Based on the Technology Acceptance Model. Sensors. 2016 Dec 17;16(12):17. doi: <https://dx.doi.org/10.3390/s16122172>.

149. Pedell S, Borda A, Keirnan A, Aimers N. Combining the Digital, Social and Physical Layer to Create Age-Friendly Cities and Communities. International Journal of Environmental Research & Public Health. 2021 01 05;18(1):05. doi: <https://dx.doi.org/10.3390/ijerph18010325>.

150. Rush KL, Singh S, Seaton CL, Burton L, Li E, Jones C, et al. Telehealth Use for Enhancing the Health of Rural Older Adults: A Systematic Mixed Studies Review. Gerontologist. 2021 Oct 18;18:18. doi: <https://dx.doi.org/10.1093/geront/gnab141>.

151. Sheats J, Winter S, Romero P, King A, Sheats JL, Winter SJ, et al. FEAST: Empowering Community Residents to Use Technology to Assess and Advocate for Healthy Food Environments. Journal of Urban Health. 2017;94(2):180-9. doi: 10.1007/s11524-017-0141-6.

152. Shijun Z, Eun-Shim N, Resnick B, Friedmann E, Brown C, Jumin P, et al. The Moderated Mediating Effect of Self-Efficacy on Exercise Among Older Adults in an Online Bone Health Intervention Study: A Parallel Process Latent Growth Curve Model. Journal of Aging & Physical Activity. 2017;25(3):378-86. doi: 10.1123/japa.2016-0216.

153. Siriaraya P, Ang CS, Bobrowicz A. Exploring the potential of virtual worlds in engaging older people and supporting healthy aging. Behaviour & Information Technology. 2014;33(3):283-94. doi: 10.1080/0144929X.2012.691552.

154. Tan JYR, Nguyen TT, Tabrisky A, Siedle-Khan R, Napoles AM. Mobile technology for healthy aging among older HIV-positive black men who have sex with men: Qualitative study. JMIR Aging. 2018;1(2). doi: 10.2196/11723.

155. Aure CF, Kluge A, Moen A. Promoting dietary awareness: Home‐dwelling older adults' perspectives on using a nutrition application. International Journal of Older People Nursing. 2020;15(4):1-10. doi: 10.1111/opn.12332.

156. Bakas T, Sampsel D, Israel J, Chamnikar A, Bodnarik B, Clark JG, et al. Using telehealth to optimize healthy independent living for older adults: A feasibility study. Geriatric Nursing. 2018;39(5):566-73. doi: 10.1016/j.gerinurse.2018.04.002.

157. Brusoski M, Rosen D. Health Promotion using Tablet Technology with Older Adult African American Methadone Clients: A Case Study. Journal of Technology in Human Services. 2015;33(2):119-32. doi: 10.1080/15228835.2014.989297.

158. Cruz M, Kugel JD. Perceptions of Older Adults on the Use of an Interactive Video Game in Promoting Health and Well-Being. Open Journal of Occupational Therapy (OJOT). 2018 Summer2018;6(3):1-10. doi: 10.15453/2168-6408.1490.

159. Dobbins S, Hubbard E, Leutwyler H. Looking Forward: A qualitative evaluation of a physical activity program for middle-aged and older adults with serious mental illness. International Psychogeriatrics. 2020;32(12):1449-56. doi: 10.1017/S1041610218002004.

160. Falls D, Shake M, Norris E, Arnett S, Taylor J, Crandall KJ. Bingocize®: Utilizing a mobile application to improve gait in community-dwelling older adults. American Journal of Recreation Therapy. 2018;17(2):9-19. doi: 10.5055/ajrt.2018.0156.

161. Gellis ZD, Kenaley B, McGinty J, Bardelli E, Davitt J, Ten Have T. Outcomes of a Telehealth Intervention for Homebound Older Adults With Heart or Chronic Respiratory Failure: A Randomized Controlled Trial. Gerontologist. 2012;52(4):541-52. doi: 10.1093/geront/gnr134.

162. Gomes GCV, Simões MdS, Lin SM, Bacha JMR, Viveiro LAP, Varise EM, et al. Feasibility, safety, acceptability, and functional outcomes of playing Nintendo Wii Fit PlusTM for frail older adults: A randomized feasibility clinical trial. Maturitas. 2018;118:20-8. doi: 10.1016/j.maturitas.2018.10.002.

163. Janhunen M, Karner V, Katajapuu N, Niiranen O, Immonen J, Karvanen J, et al. Effectiveness of Exergame Intervention on Walking in Older Adults: A Systematic Review and Meta-Analysis of Randomized Controlled Trials. Physical Therapy. 2021 09 01;101(9):01. doi: <https://dx.doi.org/10.1093/ptj/pzab152>.

164. Kampmeijer R, Pavlova M, Tambor M, Golinowska S, Groot W. The use of e-health and m-health tools in health promotion and primary prevention among older adults: a systematic literature review. BMC Health Services Research. 2016;16:467-79. doi: 10.1186/s12913-016-1522-3.

165. Kappen DL, Mirza-Babaei P, Nacke LE. Older adults' physical activity and exergames: A systematic review. International Journal of Human-Computer Interaction. 2019;35(2):140-67. doi: <https://dx.doi.org/10.1080/10447318.2018.1441253>.

166. Knight E, Petrella RJ. Prescribing physical activity for healthy aging: longitudinal follow-up and mixed method analysis of a primary care intervention. Physician & Sportsmedicine. 2014 Nov;42(4):30-8. doi: <https://dx.doi.org/10.3810/psm.2014.11.2089>.

167. Knight E, Stuckey MI, Petrella RJ. Health promotion through primary care: enhancing self-management with activity prescription and mHealth. Physician & Sportsmedicine. 2014 Sep;42(3):90-9. doi: <https://dx.doi.org/10.3810/psm.2014.09.2080>.

168. Lee J, Kim J, Jeong S, Choi H, Jin M, Kim S. A health recreation program for u-healthcare clients: Effects on mental health. Telemedicine and e-Health. 2014 Oct;20(10):930-5. doi: <https://dx.doi.org/10.1089/tmj.2013.0323>.

169. Li X, Li T, Chen J, Xie Y, An X, Lv Y, et al. A WeChat-Based Self-Management Intervention for Community Middle-Aged and Elderly Adults with Hypertension in Guangzhou, China: A Cluster-Randomized Controlled Trial. International Journal of Environmental Research & Public Health [Electronic Resource]. 2019 10 23;16(21):23. doi: <https://dx.doi.org/10.3390/ijerph16214058>.

170. O'Brien T, Meyer T. A Feasibility Study for Teaching Older Kidney Transplant Recipients How to Wear and Use an Activity Tracker to Promote Daily Physical Activity. Nephrology Nursing Journal. 2020;47(1):47-52. doi: 10.37526/1526-744x.2020.41.1.47.

171. O'Brien T, Russell CL, Tan A, Mion L, Rose K, Focht B, et al. A Pilot Randomized Controlled Trial Using SystemCHANGE TM Approach to Increase Physical Activity in Older Kidney Transplant Recipients. Progress in Transplantation. 2020 12;30(4):306-14. doi: <https://dx.doi.org/10.1177/1526924820958148>.

172. Shake MC, Crandall K, Mathews RP, Falls DG, Dispennette A. Efficacy of Bingocize: A game-centered mobile application to improve physical and cognitive performance in older adults. Games for Health. 2018 Aug;7(4):253-61. doi: <https://dx.doi.org/10.1089/g4h.2017.0139>.

173. Sowle AJ, Francis SL, Margrett JA, Shelley MC, Franke WD. A Community-Based Exergaming Physical Activity Program Improves Readiness-to-Change and Self-Efficacy Among Rural-Residing Older Adults. Journal of Aging & Physical Activity. 2017 Jul;25(3):432-7. doi: <https://dx.doi.org/10.1123/japa.2015-0278>.

174. Streber A, Abu-Omar K, Hentschke C, Rütten A. A multicenter controlled study for dementia prevention through physical, cognitive and social activities - GESTALT-kompakt. Clinical Interventions in Aging. 2017;12:2109-21. doi: 10.2147/CIA.S141163.

175. Studenski S, Perera S, Hile E, Keller V, Spadola-Bogard J, Garcia J. Interactive video dance games for healthy older adults. Journal of Nutrition, Health & Aging. 2010;14(10):850-2. doi: 10.1007/s12603-010-0119-5.

176. Thompson HJ, Demiris G, Rue T, Shatil E, Wilamowska K, Zaslavsky O, et al. A holistic approach to assess older adults' wellness using e-Health technologies. Telemedicine and e-Health. 2011 Dec;17(10):794-800. doi: <https://dx.doi.org/10.1089/tmj.2011.0059>.

177. Tollar J, Nagy F, Moizs M, Toth BE, Sanders LMJ, Hortobagyi T. Diverse Exercises Similarly Reduce Older Adults' Mobility Limitations. Medicine & Science in Sports & Exercise. 2019;51(9):1809-16. doi: 10.1249/MSS.0000000000002001.

178. Vazquez FL, Otero P, Garcia-Casal J, Blanco V, Torres AJ, Arrojo M. Efficacy of video game-based interventions for active aging. A systematic literature review and meta-analysis. PLoS ONE. 2018 Dec;13(12):e0208192. doi: <https://dx.doi.org/10.1371/journal.pone.0208192>.

179. Vizeshfar F, Ghelbash Z. Effect of a self‐care training program using smartphones on general health, nutrition status, and sleep quality in the elderly. Psychogeriatrics. 2021;21(6):910-9. doi: 10.1111/psyg.12766.

180. Wang CM, Tseng SM, Huang CS. Design of an Interactive Nostalgic Amusement Device with User-Friendly Tangible Interfaces for Improving the Health of Older Adults. Healthcare. 2020 Jun 19;8(2):19. doi: <https://dx.doi.org/10.3390/healthcare8020179>.

181. Whyatt C, Merriman NA, Young WR, Newell FN, Craig C. A Wii bit of fun: A novel platform to deliver effective balance training to older adults. Games for Health. 2015 Dec;4(6):423-33. doi: <https://dx.doi.org/10.1089/g4h.2015.0006>.

182. Wijsman CA, Westendorp RGJ, Verhagen EALM, Catt M, Slagboom PE, De Craen AJM, et al. Effects of a web-based intervention on physical activity and metabolism in older adults: Randomized controlled trial. Diabetes Technology and Therapeutics. 2015;17:S63-S4. doi: 10.1089/dia.2015.1507.

183. Krivanek T, McFeeley B, Nicastri CM, Babazadeh D, Daffner KR, Gale SA. The Brain Health Champion study: A health coaching intervention with mobile technology in older adults with mild cognitive impairment or risk factors for dementia. Alzheimer's & dementia : the journal of the Alzheimer's Association. 2021;17:e054068. doi: 10.1002/alz.054068.

184. Weakley A, Schmitter-Edgecombe M, Namboodiri S, Randhawa J, Farias ST. Piloting of a compensatory memory digital health intervention in a group of individuals with subject cognitive complaints. Alzheimer's & dementia : the journal of the Alzheimer's Association. 2021;17:e056586. doi: 10.1002/alz.056586.
