# Supplementary material for: Digital Technologies for Health Promotion and Disease Prevention in Older People: Scoping Review
Source: J Med Internet Res. 2023 Mar 23;25:e43542. doi: 10.2196/43542 (PMC10131689; doi:10.2196/43542)
Supplement: Multimedia Appendix 5 [file jmir_v25i1e43542_app5.pdf]

Use of digital offers to stay healthy and to prevent diseases among older people

Dr. Karina Karolina De Santis* (1,2), Lea Mergenthal (1), Lara Christianson (1) Annalena Bußkamp (3), Claudia Vonstein (3), Prof. Dr. Hajo Zeeb (1,2,4)

[1]Leibniz Institute for Prevention Research and Epidemiology - BIPS, Bremen; [2]Leibniz-Science Campus Digital Public Health Bremen; [3]Federal Centre for Health Education (BZgA), Cologne; [4]University of Bremen; *Email: desantis@leibniz-bips.de

Why is this study important?

- **Digital offers**, such as mobile phones, smartphones or websites can help to stay healthy and to prevent diseases
- These offers are often used by **younger** people
- It is unclear if such offers are also suitable for **independent** use by **older people**
- This study aims to find out what **digital offers** are available for older people to **keep them healthy**

How was the study done?

- The results of other scientific studies were summarized around the following topics:
  - **Older people**: Which older people use digital offers?
  - **Digital offers**: Which digital offers are used independently?
  - **Health areas**: For which health areas are the digital offers available?

Older people

Age: 50 or older	Healthy or with diseases	Have experience or need help with using digital offers

Digital offers

- Mobile phones, smartphones, electronic devices worn on the body ("wearables")
- Computer and websites
- Fitness games ("exergaming")

Health areas of digital offers

- Exercise
- Mood
- Nutrition
- Cognition

What did the study show?

- Older people **can** use and **enjoy** using digital offers for their health
- Digital offers can potentially help to **improve health**. Older people could move more, be in a better mood, eat healthier and think faster
- Digital offers should be adjusted for older people (e.g., include **larger buttons**)
- Some older people need **personal help** to use digital offers
- Digital offers can also be used to build **social contacts** with other older people

Acknowledgement: We thank Sophia Brüssermann and Kirsty Cameron for their help in preparing this document
