# Supplementary material for: Digital Technologies for Health Promotion and Disease Prevention in Older People: Scoping Review
Source: J Med Internet Res. 2023 Mar 23;25:e43542. doi: 10.2196/43542 (PMC10131689; doi:10.2196/43542)
Supplement: Multimedia Appendix 6 [file jmir_v25i1e43542_app6.pdf]

Nutzen und Nutzung digitaler Angebote zur Erhaltung der Gesundheit und Vermeidung von Erkrankungen bei älteren Menschen

Dr. Karina Karolina De Santis* (1,2), Lea Mergenthal (1), Lara Christianson (1) Annalena Bußkamp (3), Claudia Vonstein (3), Prof. Dr. Hajo Zeeb (1,2,4)

[1]Leibniz-Institut für Präventionsforschung und Epidemiologie - BIPS, Bremen; [2]Leibniz-Science Campus Digital Public Health Bremen; [3]Bundeszentrale für gesundheitliche Aufklärung (BZgA), Köln; [4]Universität Bremen; *Email: desantis@leibniz-bips.de

Warum ist diese Studie wichtig?

- **Digitale Angebote**, wie Handys, Smartphones oder Webseiten können helfen, um die Gesundheit zu erhalten und Erkrankungen zu vermeiden
- Häufig werden diese Angebote von **jüngeren** Menschen genutzt
- Es ist unklar, ob solche Angebote auch für **ältere Menschen** zur selbstständigen **Nutzung** geeignet sind
- Diese Studie möchte herauszufinden, welche **digitalen Angebote** es für ältere Menschen gibt, damit sie **länger gesund bleiben**

Wie wurde die Studie durchgeführt?

- Die Ergebnisse aus wissenschaftlichen Studien wurden in folgenden Bereichen zusammengefasst:
  - **Ältere Menschen**: Welche älteren Menschen nutzen digitale Angebote?
  - **Digitale Angebote**: Welche digitalen Angebote werden selbständig genutzt?
  - **Gesundheitsbereiche**: Für welche Gesundheitsbereiche existieren digitale Angebote?

Ältere Menschen

- Alter: 50 oder älter
- Gesund oder mit Erkrankungen
- Haben Erfahrung mit oder brauchen Hilfe, um digitale Angebote zu nutzen

Digitale Angebote

- Handys, Smartphones, am Körper getragene elektronische Geräte (“Wearables”)
- Computer und Webseiten
- Fitnessspiele (“Exergaming”)

Gesundheitsbereiche der digitalen Angebote

- Bewegung
- Stimmung
- Ernährung
- Wahrnehmung

Was hat die Studie gezeigt?

- Ältere Menschen **können** digitale Angebote für ihre Gesundheit nutzen und haben auch **Spaß** daran
- Digitale Angebote haben das **Potenzial, die Gesundheit zu verbessern**. Somit könnten sich ältere Menschen mehr bewegen, bessere Laune haben, gesünder essen und schneller denken
- Digitale Angebote sollten an ältere Menschen angepasst werden (z. B. **größere Tasten** enthalten)
- Einige ältere Menschen brauchen **persönliche Hilfe**, um die digitalen Angebote zu nutzen
- Digitale Angebote können auch benutzt werden, um **soziale Kontakte** mit anderen älteren Menschen aufzubauen

Danksagung: Wir danken Sophia Brüssermann und Kirsty Cameron für ihre Hilfe bei der Erstellung dieses Dokuments
